# Advancing SERS as a quantitative technique: challenges, considerations, and correlative approaches to aid validation

**DOI:** 10.1186/s40580-024-00443-4

**Published:** 2024-08-17

**Authors:** Sian Sloan-Dennison, Gregory Q. Wallace, Waleed A. Hassanain, Stacey Laing, Karen Faulds, Duncan Graham

**Affiliations:** Department of Pure and Applied Chemistry, Technology and Innovation Centre, University of Strathclyde, 99 George Street, Glasgow, G1 1RD UK

**Keywords:** SERS, SESORS, Quantitative, Quantification, Reproducibility, Correlative techniques, Assay platforms

## Abstract

Surface-enhanced Raman scattering (SERS) remains a significant area of research since it’s discovery 50 years ago. The surface-based technique has been used in a wide variety of fields, most prominently in chemical detection, cellular imaging and medical diagnostics, offering high sensitivity and specificity when probing and quantifying a chosen analyte or monitoring nanoparticle uptake and accumulation. However, despite its promise, SERS is mostly confined to academic laboratories and is not recognised as a gold standard analytical technique. This is due to the variations that are observed in SERS measurements, mainly caused by poorly characterised SERS substrates, lack of universal calibration methods and uncorrelated results. To convince the wider scientific community that SERS should be a routinely used analytical technique, the field is now focusing on methods that will increase the reproducibility of the SERS signals and how to validate the results with more well-established techniques. This review explores the difficulties experienced by SERS users, the methods adopted to reduce variation and suggestions of best practices and strategies that should be adopted if one is to achieve absolute quantification.

**Graphical Abstract:**

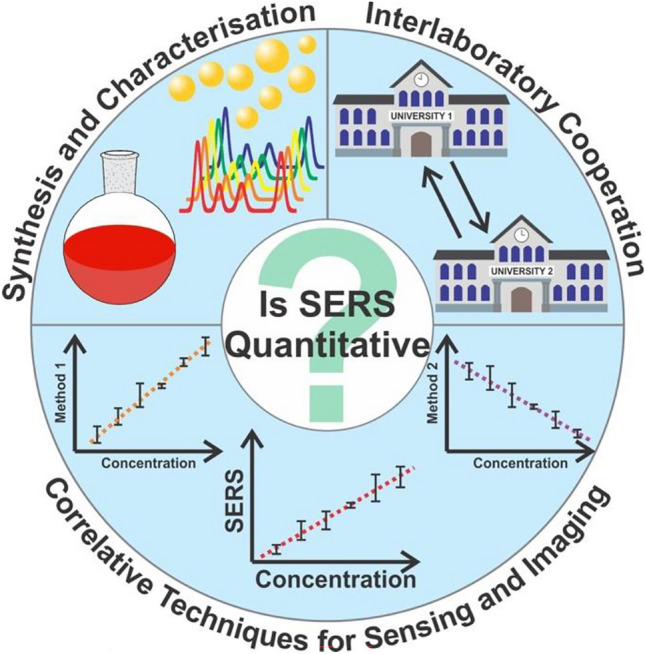

## Introduction

Surface-enhanced Raman scattering (SERS) is an analytical technique that holds tremendous promise due to the sensitive and quantitative information it can provide [1–3]. In the 50 years since its discovery, it has been applied to various research areas including chemistry, physics, materials and biomedicine [4–7]. However, additional efforts are still needed before it is accepted as a routinely used analytical technique and used in commercial products. To convince people outside the plasmonics community of the potential of SERS, leading experts in the field proposed a top 10 list of goals [8]. This included integrating classical and quantum methodologies and using modelling tools to determine Raman spectra accurately. However, most of the list focused on methods that could improve the reliability of SERS measurements via substrate fabrication, rational design of SERS substrates for targeting applications, careful characterisation of SERS substrates, and the development of standardised protocols for analyte quantification. The lack of reliability observed in SERS experiments continues to hamper its progression. A huge portion of the research in this field now focuses on methods to increase the reproducibility of SERS measurements, proving that it can indeed be reliable [9–12]. This review assesses the recent advancements that have been made in transitioning SERS from a qualitative technique to a quantitative one. First, we discuss the challenge of reliability and reproducibility that often plague the preparation and characterisation of SERS substrates and the strategies that can be employed at the nanoscale level to aid in quantification. An emphasis is also placed on interlaboratory studies that highlight how these challenges can be addressed collectively and collaboratively. Subsequent sections then highlight examples of the use of quantitative SERS across a variety of areas, notably assay development and imaging. In SERS-based assays, the objective is to quantify the presence of an analyte of interest, either by direct or indirect methods. Although the same concepts can also be applied to imaging, this review instead focuses on how SERS can be used to aid in quantifying nanoparticle uptake and how to ascertain the distribution of the nanoparticles in two- and three-dimensional biological samples. Throughout these sections, the importance of correlative techniques is discussed in the context of offering ways of validating the quantitative data obtained using SERS. Overall, this review offers necessary insights into potential best practices and new strategies that can, and should be, applied to the next generations of SERS studies to demonstrate the quantitative capabilities of SERS.

## Improving reliability and reproducibility

SERS can produce quantitative information used to determine the concentration of target analytes. The simplest method to achieve this is to build calibration curves that take the intensity or area of a well-defined peak in the SERS spectra and plot it against the concentration of the target analyte present [3]. SERS detection methods include both direct and indirect detection. In direct detection, the observed SERS spectrum is of the analyte of interest, whereas in indirect detection, the SERS spectrum of a known molecule adsorbed onto the surface of a SERS substrate is observed, and the change in this signal in response to the target analyte is instead used to generate the calibration curve. Both approaches have been applied in various assay platforms, and quantitative information on a multitude of analytes, with fantastic sensitivities, has been achieved. However, it can be challenging to replicate experimentally and build calibration curves with similar sensitivities and linearities between laboratories or even in the same laboratory with the same materials and methods. The reasons behind the poor reproducibility have been discussed at length in the literature. It is agreed that the variation in the SERS signal is due to differences in Raman spectrometer setups and lack of reproducibility in SERS substrates [1, 2, 13]. The first part of this review addresses how these parameters affect the reproducibility of SERS measurements and how standardising procedures, complete characterisation of substrates and inclusion of internal standards can minimise variation and improve the quantification of analytes.

### Interlaboratory studies

**Fig. 1:**
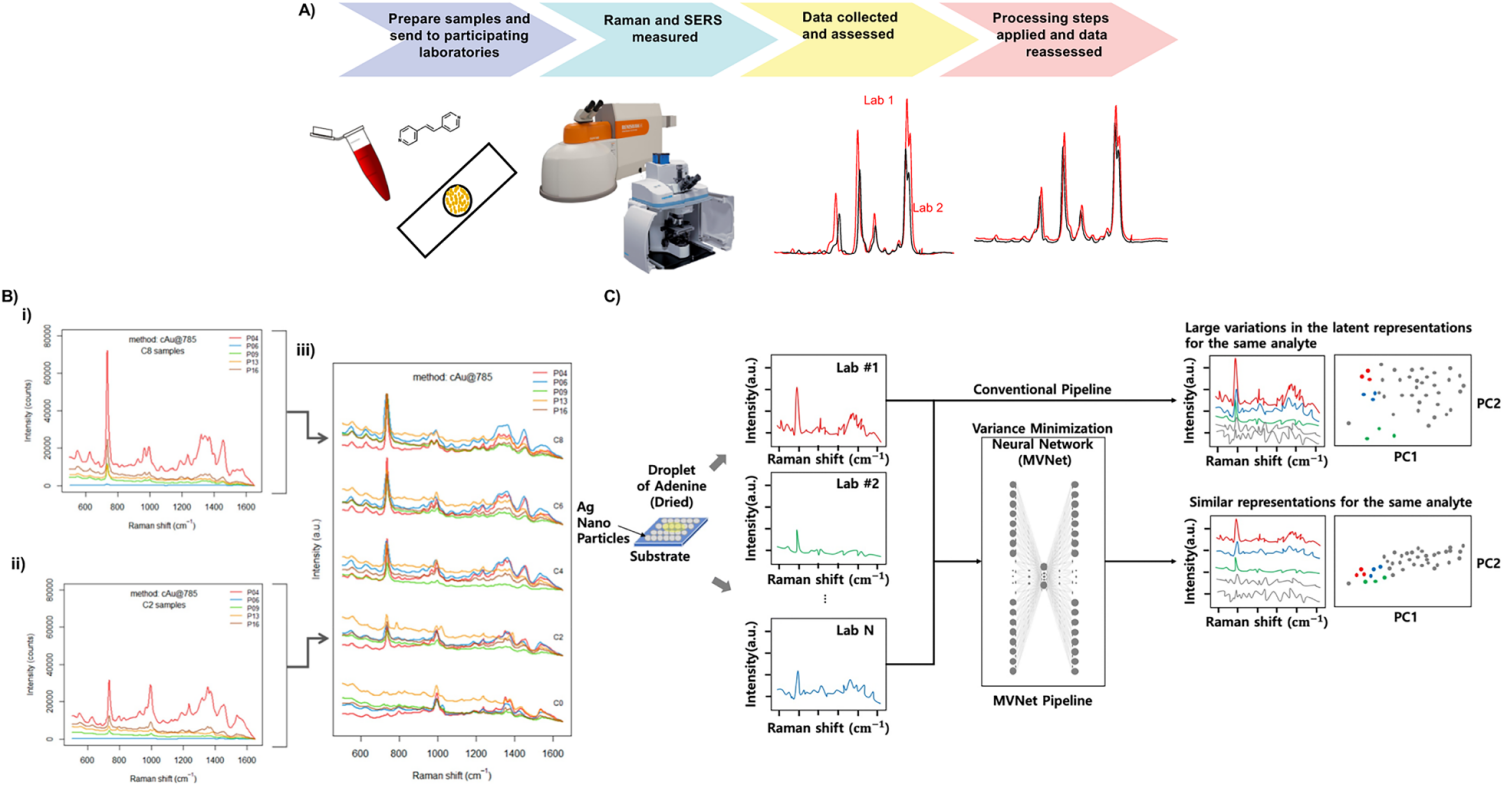
**A** Methodology of interlaboratory studies. First, samples, which include SERS substrates and analytes, are prepared by a lead laboratory and sent to participant groups. The groups measure the SERS or Raman spectra using their Raman spectrometer setup up and the results are collected and assessed by the lead laboratory. Processing steps are then applied to decrease variation. **B** Results of SERS interlaboratory study comparing the raw SERS spectra of adenine on a colloidal gold SERS substrate measured using a 785 nm laser excitation (cAu@785). The spectra shown were collected by different participating labs (P04, P06, P09, P13, and P16) for 2 different concentrations C8 (i) and C2 (ii). The spectra of five concentration levels (C0, C2, C4, C6, C8) for the cAu@785 method are shown after pre-processing, offset for clarity (iii). Adapted with permission for ref. [18] Copyright (2020) American Chemical Society. C) Schematic of the proposed MVNet pipeline. First, the SERS spectra of adenine from interlaboratory were assessed and a conventional pipeline used to evaluate the large variations. MVNet was then applied to obtain more complex, reliable, consistent and, accurate information [19]

Raman and SERS measurements are notoriously difficult to replicate between academic laboratories with factors such as Raman spectrometer setups, standard operating procedures (SOPs), and the user’s skill being linked to the variety of results achieved. Such variation among laboratories discredits SERS as a quantitative technique, and streamlined approaches are needed. To address this, interlaboratory studies have been performed with the approach aiming to improve new and existing methods and estimate precision and accuracy while assessing for potential bias [14]. There are a few examples of Raman and SERS interlaboratory studies, which have provided feedback to help harmonise and increase the reproducibility of measurements between laboratories. The general methodology of an interlaboratory study is shown in Fig. 1A.

A study performed by Raman4Clinics focused on the reproducibility of measurements on variable configurations of Raman spectroscopy platforms [15]. The interlaboratory study investigated 35 Raman spectroscopic devices with different configurations, and the results showed huge variations in peak shifts, intensities, peak widths, and noise levels. An ideal measurement would produce a zero shift, and the peak would match the theoretical values. However, this was not the case and significant variations were observed. To improve this, a wavenumber calibration was applied using paracetamol as the standard material, and a calculated calibration was devised based on the deviation between the measured and the theoretical positions of the well-defined bands. When this was applied to paracetamol and polystyrene, the peak shifts were reduced closer to zero, demonstrating it is possible to decrease the variation across instruments. To produce quantitative information from Raman and SERS measurements that are consistent between laboratories, the authors recommend the following: (i) manufacturers make full technical details of calibration corrections open access, (ii) scientists make their data openly available including the raw (unprocessed) data, and (iii) broader cooperation on the same scientific question to come up with ‘global’ solutions and reduce variations in setups used in different research groups answering the same questions. Further interlaboratory studies have also been carried out. Raman measurements of chemical vapour deposition of grown graphene were analysed by 17 groups, and significant outliers were reported, with the relative differences noted in the peak intensity ratio of up to 200% [16]. To reduce this, the study used relative intensity calibration, consistent peak fitting and data analysis, to reduce the large variations, allowing more reproducible and comparable Raman measurements across the community. Another example of an interlaboratory Raman study used Raman spectroscopy to analyse blood lymphocytes [17]. The homogeneity of scores was determined using a chi test, and the scores were analysed using an analysis of variance (ANOVA) test. Overall, good agreement between laboratories was observed, but the authors note that more research is needed to develop and verify the methodology.

A highly significant example that investigated the issues surrounding quantitative SERS measurements is Raman4clinics’ large-scale European multi-instrument interlaboratory study that was published in 2020 [18]. The study aimed to address two key questions; 1) given the simplest conditions, can a quantitative SERS method be consistently implemented by different laboratories, and 2) if different SERS methods are used to quantify the same analyte, which is the best way to compare them? Simply put, when SERS is used in different laboratories, using different instrumental setups, how reproducible and truthful are the results? 15 labs, with 6 different Raman setups, were sent a kit containing the necessary materials to prepare samples (a calibration and test set) and SERS substrates for their measurements. Adenine was selected as the analyte due to its stability, nontoxicity, and affinity for gold and silver substrates. The SERS spectra obtained from one of their tests, adenine on a colloidal gold SERS substrate analysed using a 785 nm laser excitation, are shown in Fig. 1B. The ring breathing mode of adenine is observed in the range between 715 and 750 cm^−1^ and was well resolved. However, there was a significant variation in the signal intensity for the same sample concentration, which was still present after pre-processing. This once again demonstrates the lack of reproducibility in SERS measurements across labs. A selection of substrates and laser excitations were tested, and varying results were obtained in terms of reproducibility. The most successful parameters produced an average square error of prediction (SEP) as low as 12%. However, even this low SEP did not meet the criteria for a quantitative measurement (1/SEP > 15). The results concluded that differences in the Raman microscopes account for some of the variation. Still, the biggest challenge on the route to reproducible quantitative SERS measurements is the SERS substrates themselves. Supervised learning methods have also been applied to reduce interlaboratory variation in SERS measurements. Park et al*.* proposed a data-driven solution to minimise intra and interlaboratory variations and evaluated their model against several well-known metrics such as root mean square predications and coefficient determination [19]. Their minimum-variance network (MVNet) reduced interlaboratory variability for the same target in quantitative SERS measurements and provided sufficient variability to fit a linear regression model, improving performance. Their concept is shown in Fig. 1C.

These interlaboratory studies have highlighted the need for transparency between groups and the fact that we, the spectroscopists, can account for variation in quantification by standardising procedures around the use of Raman spectrometers. This includes using similar calibration SOPs and data processing methods. The generation of spectral repositories, where spectra are openly accessible will also help further interlaboratory studies and cooperation. Similarly, given that processing the spectra is typically a precursor step for subsequent analyses, such as deep or machine learning, the algorithms used to process the spectra need to also be readily accessible. Several open-source programs are now available to process SERS spectra, allowing for this aim to become more feasible [20–22]. Furthermore, programs such as Raman2imzML [23], offer the capability of representing SERS data in other interfaces, including mass spectrometry imaging [24], that non-spectroscopists may be more familiar with. More developments are needed to help to establish the robustness of these programs, especially when the output needs to be further studied by other advanced methods. However, ‘fixing’ this is not enough to produce quantitative SERS measurements, as the most common issues that discredit quantitative SERS platforms arise from variation in the SERS substrates.

### Characterisation of SERS substrates

SERS metallic substrates can be classified into three groups. The first is solid substrates prepared using top-down approaches such as lithography. The second is nanoparticles that are immobilised onto a solid substrate, creating a film, in a bottom-up approach, and the third is nanoparticles in suspension. Each comes with advantages and disadvantages when used for quantitative measurements. For example, top-down substrates are well-defined, and use sophisticated nanofabrication methods. Therefore, it is easy to create a uniform surface with reproducible features. However, the process of fabricating the structures requires significant skills with the equipment, the fabrication is both time and cost prohibitive making scaling up a challenge, and the surfaces can have issues with spoiling. Films and suspensions are easy and cheap to produce, with the ability to scale up the synthesis. However, they are not well-defined, and experience issues with stability and unwanted aggregation. The lack of reproducibility in SERS substrates is an ongoing problem which is difficult to resolve. This is illustrated by a study by McLaughlin et al*.* [25], who used a flow cell device for in situ aggregation of silver nanoparticles. They found that when using the same nanoparticle batch, they achieved good linearity for the detection of mitoxantrone. However, when different batches of nanoparticles were used, the calibration slopes differed by up to 60% despite all experimental conditions being consistent. The SERS substrate and its controlled aggregation, therefore, has a significant impact on the signal, and we must address why this is and how it can be improved.

When selecting a SERS substrate for quantitative measurements, it should first be fully characterised. This was emphasised by Natan et al*.* who noted the importance of metrics in SERS substrate evaluation, including those relating to sensitivity, uniformity, reproducibility, as well as longevity [26]. Scanning electron microscopy (SEM) can be used to characterise solid substrates and provide valuable information on the structures and their uniformity. To investigate nanoparticles in suspension, transmission electron microscopy (TEM) is regularly used to obtain information on the nanoparticle size distribution and to assess if they are monodispersed. However, electron microscopies typically only provide snapshots regarding the size and shape distributions. Though it is possible to provide statistical information by imaging large numbers of particles, this is often not the case as it becomes time and cost prohibitive. Dynamic light scattering (DLS) and ultraviolet/visible (UV/Vis) spectroscopy are also readily used to assess the stability and aggregation state of nanoparticles. This helps us to understand the factors governing the behaviour of the nanoparticles to ensure effective formation and control of aggregation [27]. Whereas electron microscopies typically only provide snapshots regarding the size and shape distributions of a small number of nanoparticles, DLS and UV/Vis spectroscopy analyse the bulk of the sample. Whether one agrees with it or not, another method often used to characterise SERS substrates is their enhancement factor (EF).

The SERS EF represents the increase in the Raman signal expected from a molecule on a SERS substrate. The intensity of the SERS signal (I_SERS_) from a known number of molecules (N_SERS_) is measured along with the spontaneous Raman signal intensity (I_Raman_) from a known number of molecules (N_Raman_) and is most commonly calculated using the following equation:

$$\text{EF}=\frac{{I}_{SERS}/{N}_{SERS}}{{I}_{NR}/{N}_{NR}}$$

Assessing the enhancement factor of a substrate will provide an estimation of the efficiency of the substrate, as well as its sensitivity and the limit of detection achievable. When assessing the EF of a new substrate, it is important to compare it to a well-defined commercial substrate. For example, solid SERS substrates could be compared to Silmeco substrates and colloidal nanoparticles to BBI nanoparticles [28, 29]. This gives confidence that the SERS substrates are performing to a high standard. When using EF to compare substrates and assess their performance, there are a couple of things to note. For example, each measurement should be conducted under the same conditions, with the same analyte and surface coverage, and the analyte should also have no resonance contributions. One must remember that the EF will vary greatly when different analytes are used based on the interaction of the analyte and the SERS substrate. It is, therefore, very important to select the right probe. There is also a lot of ambiguity surrounding the results of EF values, which can be as low as 10^–2^ (most probably quenching) and as high as 10^15^ (most likely resonance contributions or issues with the estimations used). This needs to be clarified and more manageable for newcomers to the field. We, therefore, need to understand how to best quantify SERS EFs. Le Ru has proposed developing better ‘SERS EF standards’ that the community can refer to when estimating the EF [30]. To assess the reproducibility, interlaboratory studies would need to be carried out, and detailed SOPs would need to be developed, which would include how to measure under ‘well-defined normal conditions’. This is achievable but will require a concerted effort from the SERS community.

Although characterisation of the substrates is essential, it should not be the only parameter we consider before use. As stated by Bell et al*.* in their quantitative SERS review [13], we should ask ourselves, where is the SERS signal coming from? We know that SERS signals are dominated by molecules located in very small regions with extremely high local electric fields, also known as hotspots. However, putting this into practice, it means that only 0.59% of a Ag sphere dimer, i.e. the hotspot, contributes to 80% of the SERS signal [31]. We must, therefore, remember that the SERS signal we are measuring is only an extremely low amount of the total adsorbed molecules and is very dependent on hotspot formation. This also has huge ramifications for the reproducibility of the SERS signals. For example, one molecule located in the hotspot will have an EF in the region of 10^6^, which is just as intense as either 10^6^ molecules in solution or 10^5^ molecules in an area with an electric field enhancement of 10 [32]. Hence, small changes in the number of molecules in the hotspot will produce large variations in the signal. Aggregation or hotspot formation can be very unstructured, particularly in colloidal suspensions, which makes it very challenging to predict the EF or achieve reproducible results when different batches or even the same batch of nanoparticles are used. Bell suggests that characterisation should, therefore, include, or even focus on, targeting hotspots with the molecule of interest to increase reproducibility and correlate them with theory and simulations.

**Fig. 2:**
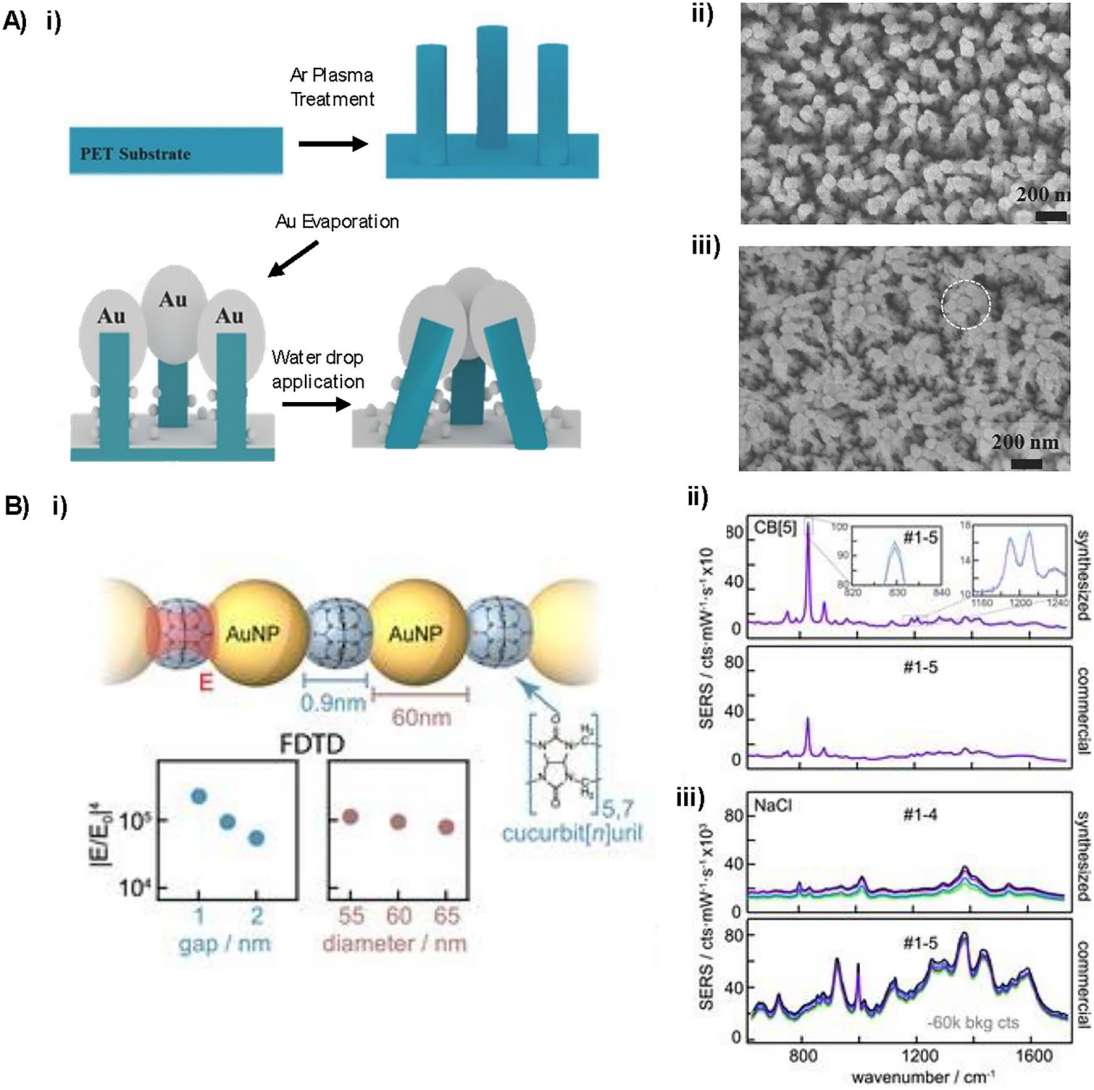
**A** i) Schematic illustration of the fabrication of the plasmonic nanopillar array. A maskless argon (Ar) plasma treatment was applied to a polyethylene terephthalate (PET) surface to create polymer nanopillars. A thick 100 nm layer of gold (Au) was then thermally evaporated onto the nanopillar to form plasmonic nanostructures. Finally, a water droplet was added to the plasmonic nanopillar array to induce capillary force-induced self-clustering. SEM images of ii) the upright plasmonic nanopillar array and iii) the self-clustered nanopillar array after water evaporation. Copyright 2017 Wiley. Used with permission from ref. [35]. **B** i) Schematic representation of CB[n] aggregated gold nanoparticles (60 nm) with 0.9 nm separation and finite-difference time domain (FDTD) simulations of the SERS enhancement as a function of gap and gold nanoparticle size. ii) SERS spectra comparing lab synthesised (top) and commercial (bottom) gold nanoparticles aggregated with CB[n] iii) SERS spectra of salt induced aggregated gold nanoparticles lab (top) and commercial (bottom).Reprinted from ref. [37]. Copyright 2020 The Authors

Solid SERS substrates exhibit dense hotspots, high stability and controllable morphology. However, their reproducibility in SERS measurements can still be increased by controlling the nanogaps between plasmonic nanomaterials. This can be achieved by modifying the synthesis. Zhao et al*.* controlled the nanogap on a SERS substrate to sub 10 nm scale by combining photolithographic metal patterning, swelling induced nano-cracking and metal spluttering (gold or silver) to create a fabricated nanogap array with an EF of 10^8^ [33]. Nanogap size can also be controlled by modifying the pressure, heating time, and heating temperature during fabrication [34]. Another method to increase reproducibility in the SERS measurements is to position the probe molecule at the hotspot on the surface [35]. By using a high aspect ratio plasmonic nanopillar array with a controlled surface energy and wetted areal fraction of 50%, capillary force-induced self-clustering of the pillars occurred, allowing for molecule enrichment atop the clustered pillars, producing a highly sensitive and reproducible solid SERS substrate. This fabrication of the pillars and SEM images before and after clustering is shown in Fig. 2A.

Controlling the aggregation of nanoparticles in suspension, as a method of increasing the reproducibility in SERS measurements, has also been investigated. Experimentally, this can be achieved by simply increasing the vortex time during the aggregation step, which has been reported to increase the formation of reproducible metal clusters, reducing the effects of random collision governed by natural convection [36]. The aggregation of gold nanoparticles has also been controlled using linker molecules, such as cucurbit[n]uril (CB[n]), which bind to gold nanoparticles to produce fixed interparticle spacing [37]. It was reported that by controlling the aggregation of the nanoparticles, the relative standard deviation between SERS measurements can be reduced to below 1%. A schematic of CB [n] induced aggregation and the reproducible SERS signal obtained from gold nanoparticles synthesised in an academic laboratory vs those commercially bought is shown in Fig. 2B. The SERS signal was also compared to gold nanoparticles aggregated with salt and indicated that CB[n] had superior reproducibility. CB[n] induced aggregation has been used to improve the quantification of target analytes in wastewater-based epidemiology [38], and for uric acid detection [39].

Another method to increase reproducibility in measurements is to control the aggregation of nanoparticles and then ‘cap’ them in a protective shell. An example of this by Braun et al*.* used molecular linkers to create stable SERS active ‘nanocapsules’ [40]. In this approach, silver nanoparticles were cross-linked with either 4-aminobenzenethiol or 1,6 hexamethylenediamine to form dimers or small clusters and then coated in a polymer that quenched the aggregation process. The Raman reporter was then infused through the polymer coat and displaced the linker molecule present at the hotspot. The SERS enhancement of the resulting nanocapsules was 300 × greater than single nanoparticles and showed a higher degree of reproducibility between batches. They were further used in Raman mapping experiments to determine the local pH in live cells [41]. Silica capping is also routinely used to quench aggregation and provide a complete shell that protects the nanoparticle and Raman reporter from the environment. The resulting stable and reproducible SERS active nanocapsules have been used in many platforms, including the quantification of SARS-CoV-2 protein with a limit of detection (LOD) of 0.01 ng/μL being achieved [42], the evaluation of nanoparticle localisation in glioblastoma multicellular tumour spheroids [43], as imaging agents for spatially offset Raman scattering studies (SORS) [44], and in photothermal applications [45].

As discussed, through careful characterisation and control of the SERS substrate and its aggregation, its sensitivity, and reproducibility can be assessed and improved. These are both important factors when selecting a SERS substrate for quantitative measurements and will help increase performance.

### Internal standards

A final method used to improve quantitative SERS measurements is the addition of an internal standard (IS). If an IS is included, it will appear consistently in the SERS spectra, and it can be used to compare and standardise the signal across many measurements and at different concentrations of target analytes to improve quantification. There are three different approaches to using ISs in SERS measurements: (1) the IS molecule is added to the solution, and its bulk Raman spectrum is measured at the same time as the SERS spectrum of the analyte; (2) the IS molecule binds to the SERS substrate and generates SERS signals that are spectrally different from the analyte; (3) the IS molecule binds to the SERS substrate and is structurally similar to those of the analyte or isotopically labelled forms of the analyte [46]. In the first approach, the IS is simply added, in a known concentration, to the solution being analysed. Its primary use is, therefore, assessing the performance of the Raman spectrometer over time. This method has been regularly used in microfluidic devices and can be used in bulk measurements to reduce variation between them to a considerable extent [47, 48]. An example of this in practice has been reported by Lee et al*.* who used acetonitrile as an IS, due to its strong band in a non-interference spectral region, in a microfluidic device designed for the SERS detection of malachite green [49]. When the samples were analysed, a calibration curve was built using the strongest SERS peak of malachite green normalised to the acetonitrile peak, producing a linear range over the 0–100 parts per billion (ppb) with a correlation coefficient R = 0.993. Ethanol has also been used as the IS to normalise SERS signals of various absorbents on Ag hydrosols prepared via laser ablation [50].

**Fig. 3:**
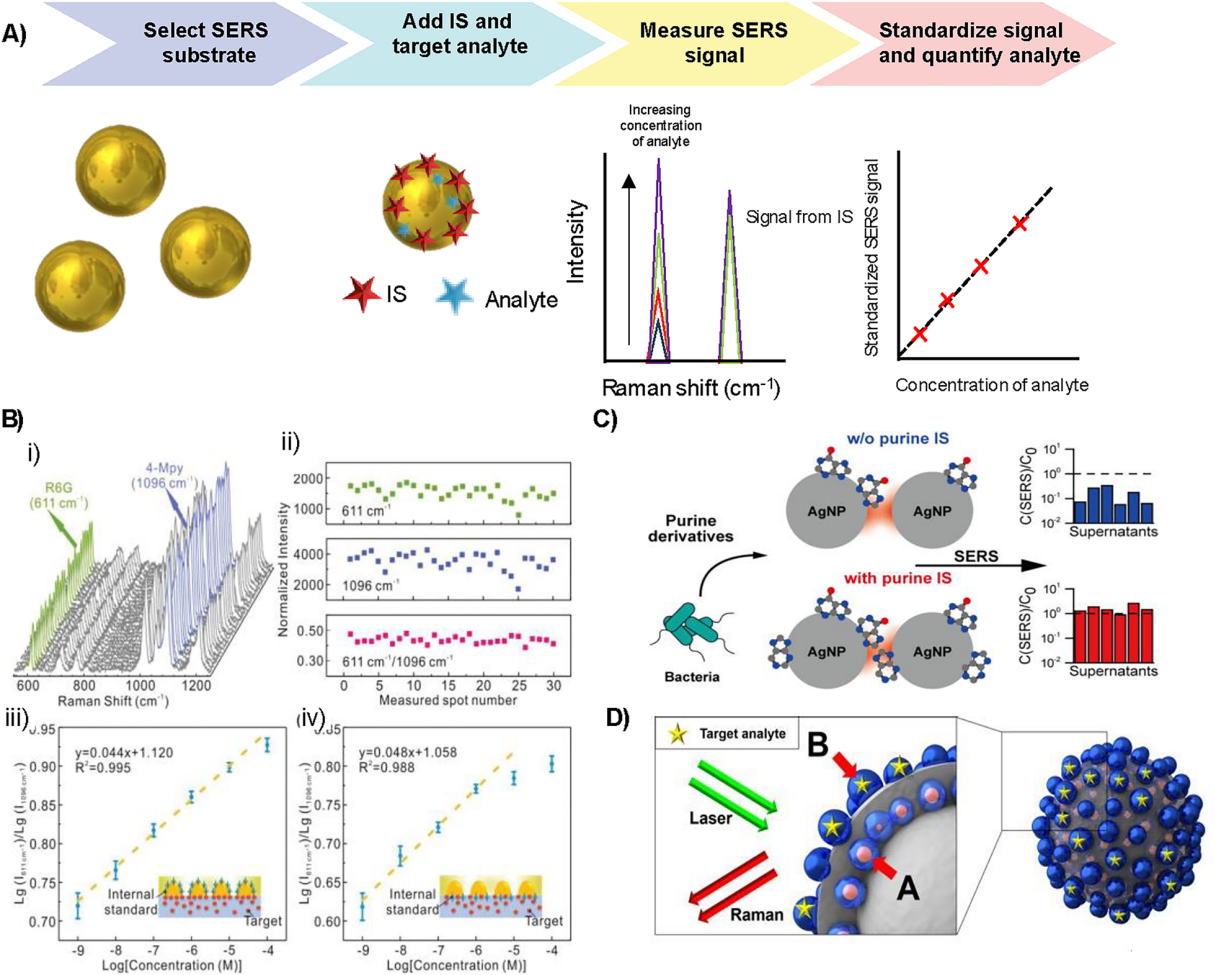
**A** Methodology for using an IS in SERS measurements. First, the SERS substrate is selected, in the example it is gold nanoparticles. The IS is then added to the substrate at a known concentration, followed by addition of the target analyte. The SERS signal at different concentrations of the target analyte is measured and the IS peak will stay consistent, while the rest of the spectrum will change with analyte concentration. Finally, a ratio of the peaks can be taken to standardise the measurement and a calibration curve can be created, which can be used for quantification. **B** i) SERS spectra of rhodamine 6G (R6G) from the Janus nanoparticles which use 4-mercaptopyridine (4-Mpy) as the IS taken from 30 random sites and ii) The peak intensity of R6G at 611 cm^−1^, 4-Mpy at 1096 cm^−1^ and their intensity ratio. iii, iv) Logarithm of the intensity ratio as a function of the analyte concentrations (Log) from different configurations as depicted in the illustrations. Reprinted from ref. [51]. Copyright 2023 The Authors. **C** Bacteria, with and without purine IS, was added to silver nanoparticles and the SERS measured. The results with the IS standardisation were more reproducible than those without. Reprinted from ref. [52]. Copyright 2023 The Authors. **D** Schematic of multi-layered silver-embedded silica nanostructure showing embedded silver nanoparticle labelled with A: the internal standard and silver nanoparticles on the outer layer for detection of B: the target analyte. Reprinted with permission from ref. [53]. Copyright 2018 American Chemical Society

Although these ISs can improve quantification, they originate as Raman signals, not SERS signals, and, therefore, do not account for changes that can occur to the SERS substrate. Changes such as aggregation, spoiling or deactivation could lead to a huge change in the SERS signal, and one way to monitor this is to use an IS that is SERS active. This IS can bind to the SERS substrate and experience the same changes that affect the SERS signal of the target analyte. The general methodology for using a SERS active IS is outlined in Fig. 3A.

SERS active ISs are regularly applied to solid SERS substrates to improve the quantitative analysis. Li et al*.* incorporated a SERS active IS into a SERS substrate consisting of a polymer membrane embedded with Janus nanoparticles [51]. The curved side of the Janus particles was coated in the IS and protected by the polymer, while the flat side of the nanoparticle was exposed to the target analyte, which was adsorbed onto it for SERS measurements. Using this platform an extension in linear region for quantitative response was observed for the biochemical molecules including rhodamine 6G (R6G), crystal violet, and adenine. This is shown in Fig. 3B, along with an illustration of the substrate. A simple SERS active IS approach reported by Zhao et al*.* added urine creatinine as the IS to a solid SERS substrate, which was then used to standardise the detection of uric acid in urine [54]. This approach produced a satisfactory linear correlation, with good signal reproducibility between each substrate.

An alternative approach is to use an IS chemically similar to the target analyte. In this case, the IS will have similar binding to the SERS substrate as the analyte and should experience the same fluctuations in the SERS signal. This has been shown by Chung et al. who used purine as an IS to address the variation in signals measured from different batches of SERS substrates when detecting purine derivatives released by bacteria [52]. Purine was selected as it exhibits similar affinity to the SERS substrate as the target analytes, adenine and hypoxanthine. Therefore, it should behave in the same way to them, while still producing a SERS signal that does not interfere with their spectral peaks. The schematic in Fig. 3C illustrates the workflow of the approach. Purine was added to the supernatants of *S. aureus* and *E. coli* before addition to the surface of silver nanoparticles. When the SERS was measured and the analytes quantified, the calibration that considered the IS purine peak outperformed those without, exhibiting a tenfold increase in predictive accuracy. Quantification of the analytes using this method also correlated well with liquid chromatography–mass spectrometry results.

Another example of a SERS active IS is the use of isotope-edited internal standards (IEISs). A commonly used SERS active IEIS platform uses rhodamine 6G and its isotope [55]. An early report of this added silver nanoparticles to a mixture containing Rhodamine 6G (R6G-d_0_) and Rhodamine 6G (RGD-d_4_) edited with deuterium and when measured, bands from both were observed in the spectra and the signal could be standardised. The method demonstrated that it could correct for fluctuations in intensity caused by poor reproducibility of the SERS substrate, complex structure of the surface, distance dependence of SERS intensity as analyte concentration, and the variations in collection or excitation of the Raman system. IEISs have also been used for the quantitative detection of tryptophan and caffeine with improved linearity and reduced errors for test set predictions [56].

These IS approaches all improve quantification; however, they do have some disadvantages. For example, the IS molecules compete for surface adsorption with the target analyte or another Raman reporter, and displacement of the analyte can occur. The IS could also be influenced by the microenvironment, resulting in changes in intensity or frequency independent of the changes in the SERS substrate. An embedded IS approach using a core-molecule-shell (CMS) has been developed to overcome this. In this approach, the IS is fixed to the core nanoparticle surface which is then encapsulated in another plasmonic metal used to sense the targeting analyte. Quantitative analysis is possible as the IS corrects for signal fluctuations between samples and measurement conditions [57]. An example of this used a gold nanosphere core coated in 4-mercaptopyridine (4-Mpy), the Raman reporter acting as the IS, and cysteamine, which aided in the production of a silver shell of roughly 4.5 nm. The CMS was then used to quantitatively detect 1,4-phenylene diisocyanide (PDI). They observed that the PDI signal fluctuated synchronously with 4-Mpy, and that normalising the PDI signal to that of 4-Mpy corrected for the fluctuations in both aggregation state and instrumental factors. Another embedded IS example used a multi-layered nanostructure consisting of a silica core that was coated with silver nanoparticles functionalised with 4-bromobenzenethiol, acting as a Raman label, which was then encapsulated in a silica shell decorated with another layer of silver nanoparticles [53]. The embedded silver nanoparticles act as the IS, and the outer silver nanoparticles are used as sensor sites to detect model pesticides. This is shown in Fig. 3D. In an alternative approach, gold nanoparticles coated in 1,4-benzenedithiol (BDT) and 4-mercaptophenylboronic (MPBA) were synthesised to quantify hydrogen peroxide (H_2_O_2_) and cholesterol levels in solution and inside living and fixed cells [58]. In this design, BDT served as the embedded IS and MPBA was the Raman probe that was converted into 4-hydroxythiophenol (4-HBT) via selective oxidation by H_2_O_2_, which was present due to the oxidation of cholesterol by an enzyme. Another alternative nanoparticle system, gold nanoparticles coated with two ISs, 2-mercaptobenzimidazole (2-MB) and p-aminothiophenol (PATP), has been used to quantify phosmet residues on apple skins [59]. By applying a multiple IS method with spectral shape deformation quantitative theory, which took advantage of all spectral information of the multiple ISs, the concentration predictions were significantly more accurate and precise than a univariate ratiometric approach. Further examples that utilised embedded ISs include gold-4-Mpy-silver core–shell nanoparticles to quantify thiram [60], gold-4-MBN-silver nanoparticle (MBN, 4-mercaptobenzonitrile) for nicotine detection [61], and silver-4-MBN-silver nanoarrays for detection of deltamethrin in foods [62].

A final method to improve analyte quantification is to use a standard addition method (SAM). If we use the quantification of uric acid as an example, it can be difficult to achieve absolute quantification of it in an unknown sample as uric acid is always present in the urine. Therefore, the SAM approach can be applied by spiking in known amounts of uric acid into the urine, measuring the SERS signal, and plotting the peak area of a characteristic uric acid peak against the concentration that was spiked in. If a straight line is achieved *(y* = *mx* + *b:* where *m* and *b* are the slope of the line and y-intercept) it can then be used to determine the concentration of the uric acid initially present in the urine, by using the point at which the extrapolated line crosses the concentration axis (x) at zero signal (where *y* = *0* and the *x* = − *b/m* such that the concentration = *b/m*) [63]. Using this approach Westley et al. predicted the concentration of uric acid in 21 different patients with an average difference of only 9% when compared to high-performance liquid chromatography (HPLC) [64]. Furthermore, the analysis was carried out in triplicate using different batches of nanoparticles demonstrating excellent reproducibility. The SAM method has also been applied for the absolute quantification of nitroxoline [65], nicotine [66] and thiram [67].

Overall, a unified approach to calibrating, measuring, and processing SERS spectra is achievable by addressing the experimental parameters that affect the reproducibility of a SERS measurement between Raman spectrometers. Furthermore, complete characterisation of SERS substrates and making adaptations to the substrates during synthesis, along with the inclusion of internal standards, can help standardise the SERS signal to enable movement towards absolute quantification of target analytes.

## SERS-based quantification in assay platforms

It is possible to improve the quantification using SERS by standardising experimental parameters which improve consistency in measurements and reliability in SERS substrates. However, it can still remain a challenge to achieve quantification when SERS substrates are applied in detection assays. To address this, we can consider the different types of assay platforms used. Microfluidic devices and paper-based sensors can be seamlessly merged with SERS to create versatile, selective and quantitative detection platforms for different applications. This synergistic approach not only enhances the accessibility of SERS technology but also opens new possibilities for real-time analysis in diverse settings, ultimately facilitating quicker decision-making and improved resource allocation in critical situations. This next section will focus on the advantages of these platforms and assess their capabilities at producing reliable and quantitative results for a variety of target analytes.

Microfluidic devices are known as lab-on-a-chip (LoC) as they facilitate the performance of the required procedures for analyte detection in one rapid automated step while maintaining high precision [68]. They have the advantages of low sample volume consumption, short detection time, portability, automation and reduction of human error [69]. SERS detection can also be applied to LoC platforms to improve sensitivity, which can be achieved either by using colloidal or stationary nanostructures as SERS active substrates in the microfluidic devices. The colloidal nanostructures can be injected into the microfluidic channels and mixed with the analyte solution. However, this interaction inside the device can block the microchannels due to the aggregation of the nanostructures, resulting in SERS signal variations and poor quantification [70]. To overcome these limitations, the analyte and colloidal nanostructures can be mixed off-chip and then injected via the same port into the system [69]. Another solution is to apply the droplet microfluidics approach, which greatly minimises the blocking of channels by enabling the analyte to interact with plasmonic nanostructures inside tiny volume droplets [71, 72]. The use of solid stationary nanostructures in the microchannels offers higher precision than colloidal substrates, as the morphology of the substrates can be controlled, which further improves the sensitivity and reproducibility of SERS measurements in microfluidic devices [72].

Although the combination of SERS with microfluidic devices has improved the detection sensitivity and quantification capacity of the system, the memory effect is a major problem that limits the repeated use of the substrate [69, 72]. Therefore, microfluidic devices are mostly used for a single measurement which increases the cost of the detection. Krafft et al*.* reported the design of a disposable microfluidic device (2.5 × 2.5 cm) utilizing a polydimethylsiloxane (PDMS) nanoporous membrane for the concentration and SERS detection of bacteria (*E. coli DH5α* and *Pseudomonas taiwanensis* VLB120) in a spiked tap water sample [73]. As the concentration of pathogen microorganisms in drinking water is low (0–10^2^ CFU/mL), an efficient enrichment step was required to obtain a detectable signal. Therefore, the device was composed of two stacked perpendicular channels attached via a nanoporous polycarbonate track-etched membrane. One of the channels was held under a positive voltage and supplied a mixture of bacteria and silver nanoparticles and the second channel was grounded. This created a strong electro-osmotic flow that dragged the bacteria and Ag clusters toward the membrane, where they were sterically trapped and concentrated, enabling a rapid detection (< 2 min). In another application, Asgari et al*.* described the integration of a T-junction PDMS-based hydrodynamic flow-focusing microfluidic device with SERS for the rapid detection (1 h) of *E. coli* O157:H7 cells in food samples [74]. A selective SERS nanoprobe was anchored onto the bacterial cells and separated them from lettuce samples off-chip. The separated cells were then detected within the microfluidic device with a] LOD of 0.5 CFU/mL. Although the performance of this device was efficient, the method demonstrated some non-specific binding against another closely related bacteria strain, *E. coli* K12.

**Fig. 4:**
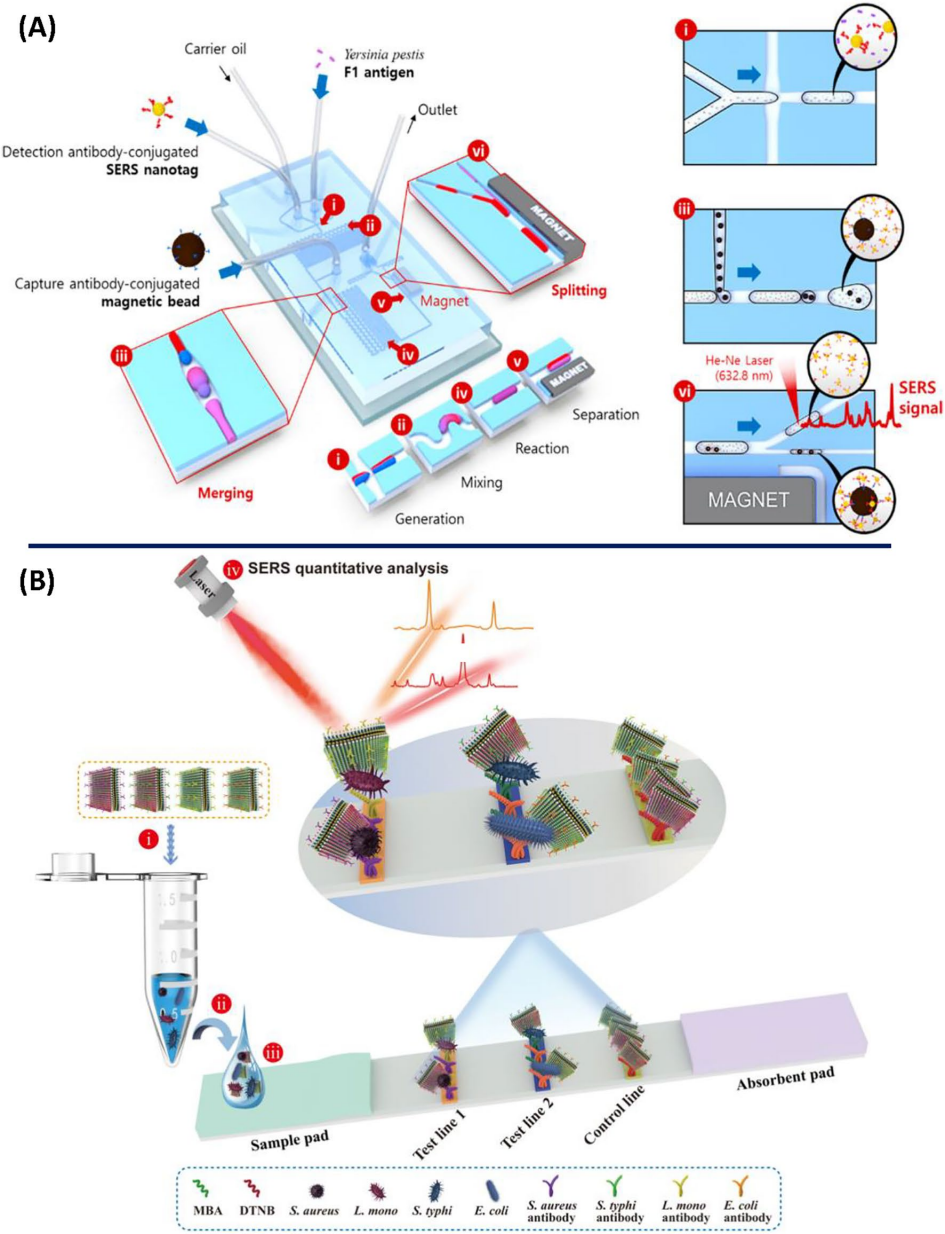
Schematic illustrations for (**A**) the integrated SERS-based microfluidic channel composed of six microdroplet compartments: (i) droplet generation, (ii) droplet mixing for the first immunoreaction, (iii) droplet merging for the formation of magnetic immunocomplexes, (iv) droplet mixing for the second immunoreaction, (v) droplet splitting for the wash-free immunoassay and (vi) Raman detection of unbound SERS nanotags in supernatant solution droplets. Extended images for (i) droplet generation, (iii) droplet merging and (vi) droplet splitting. Adapted with permission from ref. [75]. Copyright (2017) American Chemical Society (**B**) The 3D GO@Au/Ag nanosticker lateral flow strip for the multiplex SERS detection of four strains of bacteria. Reprinted from [85], copyright 2022, with permission from Elsevier

Another design of a multifunctional microdroplet-based merging and splitting microfluidic platform was reported by Choi et al*.* for the rapid SERS quantification (< 10 min) of F1 antigen in *Yersinia pestis* (Fig. 4A) [75]. The PDMS-based microfluidic device design enabled the formation of magnetic immunocomplexes through a sequence of microdroplet generation, transport and merging operations, while wash-free steps were realised through the magnetic splitting of a large droplet into two daughter droplets. The reported LOD was estimated to be 59.6 pg/mL. This multifunctional microfluidic platform enabled the performance of complex multistep immunoassays in nanolitre volumes for the safe and sensitive quantification of hazardous infectious materials. Another approach for the combination of SERS with microfluidics involves the integration of dielectrophoresis (DEP) with SERS-microfluidics in a single platform [76]. This is used to enable the rapid separation and concentration of pathogens from biological samples using non-uniform electric fields, which manipulates the movement of molecules in fluids due to their electrical properties. The resulting sample concentration and/or enrichment improves the SERS detection sensitivity. For example, a DEP-SERS barcode sensing strategy using nanoaggregate-embedded beads was described for the on-line multiplex detection of *Salmonella choleraesuis* and *Neisseria lactamica* [77]. The platform was combined with a confocal micro Raman system in a compact setup for *in-situ* bacteria detection within 10 min. The LOD was estimated to be 70 colony-forming unit (CFU)/mL. This demonstrates the strong potential of a DEP-SERS-microfluidics system to be applied, as a compact and portable platform, for the highly rapid and sensitive on-line detection of pathogens.

Paper-based sensors, including paper-based microfluidics and lateral flow strips, are another platform that integrate well with SERS. Microfluidic paper-based analytical devices (µPAD) are characterised by many advantages, such as: simple fabrication, user-friendliness, cost-effectiveness, portability and disposability. Unlike the conventional silicon/glass-based microfluidic devices, the µPAD possess a natural capillary-driven fluidic flow through cellulose fibres, thus enabling the rapid free movement of samples and reagents through the porous paper network without the need for external pushing forces, i.e., pressure pumps [78]. The most common method for their fabrication is the wax printing technique that can create hydrophobic barrier channels through the paper surface to control the fluid flow direction [68]. A recombinase polymerase amplification-integrated μPAD was fabricated for the rapid SERS detection of *Salmonella typhi* in real food samples [78]. The μPAD design contained a dumbbell shaped hydrophilic zone for the loading of a SERS conjugate in one side. After drying, the target bacterial DNA was loaded on the same side, allowed to interact with the SERS conjugate and diffuse to the detection zone. The SERS measurement was carried out within 1 min after sample addition and the reported LOD was 3–4 CFU/mL. The calculated relative standard deviation (RSD) values for repeatability and reproducibility using this μPAD design were less than 6%.

As a substrate, lateral flow test strips are currently used as a selective, stable, cost-effective and user-friendly platform for the rapid and infield detection of different targets in diverse biological matrices [68]. Unlike the conventional lateral flow test, SERS detection of the accumulated nanostructured conjugates onto the strip test line enables the ultrasensitive quantification of the target analyte without the need for a highly patterned SERS substrate. Therefore, as in microfluidics, the integration of SERS with lateral flow tests improves the test performance, in terms of the detection sensitivity and the selective quantification capacity, while maintaining the short detection time and assay simplicity [79]. Accordingly, this combination has been extensively described before as a potential alternative tool of traditional detection methods for various applications [80–84]. In a recent study, Wang et al*.* presented a 3D membrane-like nanostructured conjugate composed of graphene oxide (GO)@gold/silver (Au/Ag) nanostickers for the multiplexed ultrasensitive quantification (0–10^6^ cells/mL) of *Salmonella typhi*, *E. coli*, *S. aureus* and *Listeria monocytogenes* in a single test within 20 min (Fig. 4B) [85].

The grafting of numerous Ag satellites onto the nanostickers increased the surface area for bacterial binding, as well as the number of hotspots, thus improving the detection sensitivity. The reported LODs using the proposed lateral flow strip design were 9 cells/mL for the four pathogens in real clinical matrices (blood, urine and sputum). In a more recent application, a SERS-lateral flow biosensor was reported for the absolute quantification of serum exosomes, SKBR and MCF, as circulating biomarkers for breast cancer diagnostics and prognostics at various pathological states [86]. The combination of the SERS-lateral flow biosensor with multivariate analysis and spectral unmixing enabled the multiplexed quantification of the serum exosomes in a single test with less interference from the complex body fluids. The method reported a linear quantification in the range 0–500 × 10^7^ particles/mL with detection limits of 3.27 × 10^6^ and 4.80 × 10^6^ particles/mL for the serum SKBR and MCF exosomes, respectively.

The benefits of these different SERS-based assay platforms for the detection and quantification of various biological analytes highlight the applicability of SERS in a wide range of useful applications. This further supports the need for standardised methods of validation to warrant the adoption of the technique in clinics and commercial testing platforms, where the reliability of the analytical data is essential.

### Correlative techniques to validate SERS quantification assays

When moving SERS measurements out of the lab and into the ‘real world’, an important aspect is investigating how reliable the results are. When assessing ‘real-life’ samples using a SERS assay, we must ensure that the results achieved are credible, which can be achieved by correlating them with another, well established, technique. Having the results of both methods will help improve the reliability of the SERS platform and convince the greater community that the results are valid. Examples of how this can be achieved are: by running an enzyme-linked immunosorbent assay (ELISA) on biological samples to demonstrate that the SERS assay detects the same concentration as the commonly applied ELISA [87]; fluorescent staining of cells to indicate that the nanoparticles are targeting the correct cellular component [88]; and mass spectrometry to prove that substrates capture the correct target analyte [89]. These approaches are useful for initial validation of the method; however, in practice, this can be difficult to achieve as there may be low sample volumes, making it impossible to perform repeat analysis. Also, the gold standard techniques are often slow and not sensitive, and SERS has been implemented to improve both parameters.

To overcome this, recent efforts have focused on augmenting SERS with a range of techniques, such as chromatography, microfluidics, electrochemistry, labelling techniques, etc., enabling both measurements to be taken on the same sample and within a similar timeframe [90]. Such correlation with other analytical techniques enhances the detection capabilities, as well as enabling comprehensive characterisation and quantification of analytes in various applications, such as biomedical diagnostics, environmental monitoring, pharmaceuticals and food analysis. In the following subsections, we highlight some recent advances, within the last decade, in correlating SERS with other techniques to improve its accurate quantification capability.

#### Phase separation and chromatography

The coupling of SERS with phase separation [91–94], and chromatographic techniques, like thin layer chromatography (TLC) [95–98], and high-performance liquid chromatography (HPLC) [99–102], allows for the individual isolation of the analytes from complex mixtures before their SERS detection. This coupling enables the quantitative determination of analytes with enhanced selectivity and sensitivity.

Phase separation is a technique that is capable of isolating and concentrating a substance or similar materials from a mixture, exploiting the unique selectivity and distribution of specific analytes across different phases. Following phase separation, the analyte becomes relatively concentrated and pure; thus improving the sensitivity of the SERS detection. Solid-phase microextraction (SPME) is renowned for its ability to separate and pre-concentrate target analytes from either a gaseous or solvent phase. It has garnered popularity owing to its solvent-conserving nature and ease of automation. Zhang et al*.* described a sensor system based on heat-treatment SPME and SERS (HT-SPME-SERS) for the *in-situ* detection of isocarbophos pesticide in complex tea matrix [103]. Pesticide molecules volatilizing from solution were captured by a SERS substrate (Cupper (Cu) @ reduced graphene oxide (rGO) @Ag nanoparticles) and generated real-time SERS signals using a hand-held Raman spectrometer. The rGO films growing on the substrate adjusted the surface bore diameter of Cu foam and trapped the pesticide molecules through hydrophobic interactions and π–π interactions. The reported LOD was 0.00451 parts per million (ppm). The results obtained from this sensor system were verified by gas chromatography–mass spectrometry with a good percentage agreement between both methods. Such combination of phase separation and SERS would provide higher sensitivity, simpler operation, short analysis time and less sample consumption, therefore enhancing the capabilities of SERS detection. However, there is an apparent drawback associated with this technique, which is the lack of selectivity of some sorbents. There may be several compounds in the extracted mixture having similar chemical structures or polarities. Therefore, the obtained spectrum may include some overlapping peaks or represent only one major compound from the mixture with the strongest affinity to the metal substrate.

To remove interference from a complex mixture and increase the sensitivity of the detection, SERS could be combined with a chromatographic process prior to detection. The principle of TLC is based on different affinities of a specific compound towards the mobile and stationary phases, thus affecting the migration speed of analytes on a silica gel plate. Therefore, TLC can provide a simple process for separating chemicals on a plate in a short space of time and improving the SERS detection efficacy. However, some compounds of very similar chemical structures or polarities are still difficult to separate completely through TLC. Therefore, the SERS signal of an analyte of interest could still be obscured. Lu et al*.* reported a TLC-SERS method for the quantitative monitoring of ofloxacin in beef combined with machine learning analysis [104]. After separating the ofloxacin from a real beef juice sample on a TLC plate, the separated spot was subjected to a silver nanoparticle solution and scanned by a handheld Raman spectrometer. As a nonlinear relationship was obtained between the concentration of the analyte and the SERS signal, a principal component analysis-back propagation neural network (PCA-BPNN) model was used for the quantitative prediction of the ofloxacin concentration with a LOD of 0.01 ppm. Accordingly, this result demonstrated that TLC-SERS combined with machine-learning analysis could be used as an effective technique for quantifying antibiotics in food safety monitoring.

HPLC is considered a superior method of separation to isolate individual components from complex samples, overcoming the limitations mentioned in TLC. After HPLC separation, the effluent interacts with a SERS substrate, either on-line or at-line, for sensitive SERS detection. The complete separation of complex matrix components by HPLC helps in producing individual SERS signals. Thus, highly sensitive and selective SERS detection can be obtained. Shen et al*.* described a HPLC-SERS hyphenated system for the continuous on-line separation and structural identification of illegal additives in a hypoglycaemic supplement [105]. The additives were separated by HPLC, and the effluents were loaded onto automatically and continuously replaceable paper substrates for real-time SERS measurements. The continuous replacement of the paper SERS substrate, by a winder immediately after each pick-up of the effluent liquids, enabled the acquisition of SERS signal from each substrate. Therefore, the SERS spectrum at corresponding HPLC retention times were specifically and easily obtained. This construction enabled a high separation efficiency and a sensitive quantitative detection down to 10^−5^ mol/dm^3^.

#### Mass spectrometry

Combining mass spectrometry (MS) with SERS offers a powerful analytical tool with complementary strengths for the accurate characterisation of molecules [106–109]. This integration leverages the high sensitivity and molecular specificity of both techniques to provide a comprehensive chemical information about the target analyte. MS excels in identifying and quantifying individual molecules based on their mass-to-charge ratios, while SERS offers enhanced detection sensitivity and the ability to probe molecular structure and interactions. By coupling these techniques, a simultaneous detection and characterisation of analytes could be achieved with high specificity and sensitivity, enabling a deeper understanding of complex samples.

**Fig. 5:**
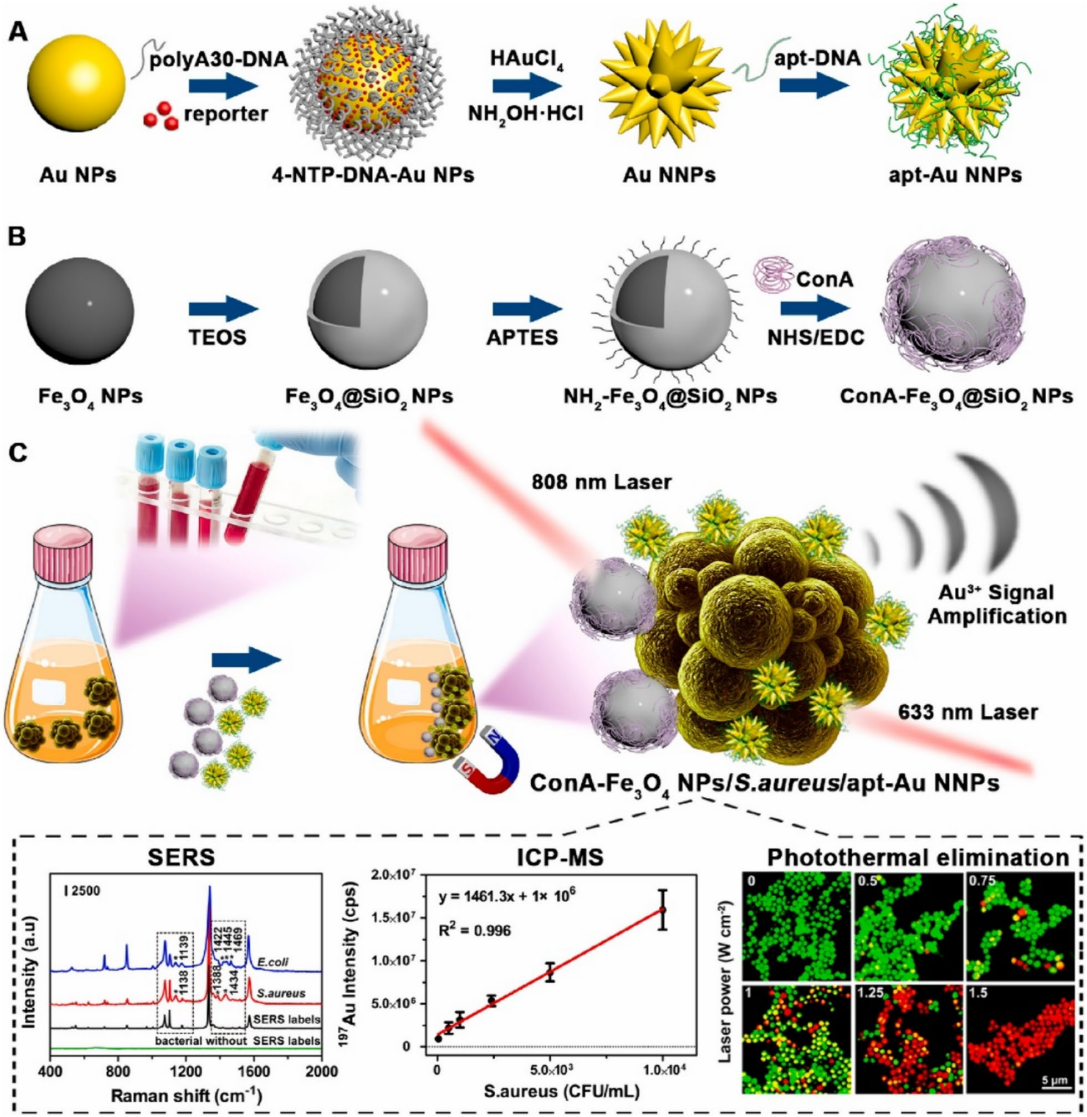
The synthetic procedures of **A** apt-Au nanogap nanoparticles (apt-Au NNPs) and **B** ConA-Fe_3_O_4_@SiO_2_ nanoparticles, and **C** the principle of multimodal bacteria detection and photothermal bacteria-elimination using Au NNP-based sandwich nanocomposites. Reprinted from ref. [110], copyright 2023, with permission from Elsevier

Huang et al*.* presented the design of multifunctional Au nano-bridged nanogap probes (Fig. 5) as inductively coupled plasma mass spectrometry (ICP-MS) and SERS dual-signal tags for bacterial discrimination, quantitative detection and photothermal bactericidal activity [110]. The nanocomposite consisted of concanavalin A (ConA)- iron oxide (Fe_3_O_4_) @silica (SiO_2_) nanoparticles for the magnetic enrichment and photothermal killing of *S. aureus* bacteria (Fig. 5B). Additionally, the aptamer (apt)-modified Au nanogap nanoparticles (apt-Au NNPs) and the ConA-Fe_3_O_4_@SiO_2_ nanoparticles were employed as dual-signal tags where a sandwich formed in the presence of complementary bacteria to enable both SERS sensing and ICP-MS quantification. The method provided a linear relationship for the bacterial quantification in the range 50–10^4^ CFU/mL with a LOD of 11 CFU/mL. The method was also applied for the bacterial detection in an actual human serum sample showing high selectivity and sensitivity. In a recent study, Fan et al. reported a liquid chromatography with tandem mass spectrometry (LC–MS/MS)-assisted label-free SERS blood analysis method for the detection of lung cancer based on using a self-positioned SERS platform, driven by hydrophilic-hydrophobic interactions (LOD = 3.98 × 10^–11^ M for R6G dye) [111]. The SERS signals from serum components with different molecular weights were analysed through a serum filtration process, and the abnormalities in the SERS spectra of the serum were confirmed by the LC–MS/MS method. Following that, machine learning algorithms were employed to explore the diagnostic information of the blood spectral data, achieving detection accuracy of 96.3% for discriminating lung cancer samples from the benign samples.

#### Electrochemistry

In the context of electrochemical (EC)-assisted SERS, there are several ways in which EC could improve the SERS sensing. For example, by augmenting selectivity through surface charge manipulation and by ensuring a controlled adsorption of analytes and/or desorption of interfering matrix components through various EC processes [112]. The alteration in the electronic distribution of adsorbed molecules, even in their ground state, can lead to various enhancements across different vibrational modes. Additionally, EC could also assist in achieving maximum substrate surface coverage with the analyte by changes in the surface orientation of the adsorbate, as well as in activating surfaces through oxidation/reduction cycles, leading to improved reproducibility of measurements [113]. Additionally, EC could amplify the SERS chemical enhancement and attract molecules closer to regions of high electromagnetic field, thus promoting the SERS electromagnetic enhancement. Moreover, cross-validation of the results becomes feasible when quantification is done by both EC and SERS [112, 114–116].

Extensive studies have previously reported the use of EC-SERS sensors in the quantification of different target analytes [112, 114, 117]. For example, Ibáñez et al*.* described the generation of reproducible Au nanostructures on a metallic screen-printed electrode (SPE) as a SERS substrate for the detection and characterisation of a family of B vitamins. The method was successful in elucidating vitamins B1, B2, B3, B6 and B12 in commercial multivitamin complexes, showing their distinct Raman spectra due to their varied chemical structures [118]. Another recyclable substrate design fabricated by electrochemical reduction was reported by Hu et al*.* for miRNA 21 detection [119]. In this method, 2′-hydroxymethyl-3,4-thylenedioxythiophene (EDOT-OH) was electropolymerized on an electrode surface to form the reduced form PEDOT-OH, which acted as a SERS substrate and as a signal probe. The substrate displayed a change in the signal intensity between its reduced and oxidised states. In the presence of the target and by combining with glucose and magnetic beads modified with enzyme-functionalised DNA, PEDOT-OH was changed from reduced to oxidised state, resulting in SERS signal reduction. When different concentrations of target were added, the amount of oxidised PEDOT-OH was different, so the quantitative detection of miRNA was realised. After use, the signal of the oxidised PEDOT-OH on the electrode surface could be restored by applying a reduction voltage for 30 s. The described SERS platform reported the detection of miRNA 21 in the range of 100 fM to 1 μM with a LOD of 0.03 fM.

Another application was presented by Lu et al*.* for the duplex detection of lung cancer biomarkers (CEA and CK-19) using an EC-SERS immunosensor [120]. Raman dyes (thionine and Nile blue A)-decorated resin microspheres demonstrated both strong distinct SERS signals and electrochemical redox characteristic peaks. Therefore, they were used for the simultaneous detection of the biomarkers using square wave voltammetry and SERS. This was carried out following the formation of sandwich structures between Raman dye-labelled gold nanoparticle coated microspheres functionalised with a primary antibody, and an electrode modified with gold nanoparticles functionalised with a secondary antibody and target antigens. As a result, a duplex immunoassay for both analytes based on multiple labels was developed using electrochemical and SERS detection. The reported LOD was 0.01 and 0.04 ng/mL for CEA and CK-19, respectively.

#### Surface plasmon resonance

In surface plasmon resonance (SPR) measurements, the interaction between the sensor surface and target analytes lead to shifts in resonance wavelength or angle, facilitating real-time monitoring and quantitative analysis. This capability enables the investigation of a wide range of biomolecular interactions, including those involving DNA [121], viruses,[122], antibodies [123] and antigens [124]. SPR offers additional advantages such as high-throughput capabilities and cost-effectiveness. However, challenges persist, including the requirement for capture layers to aid in molecular identification and limitations in achieving ultra-low LODs compared to alternative methods. Conversely, SERS is a highly specific technique capable of distinguishing between various analytes based on their unique vibrational characteristics. The electromagnetic enhancement induced by SPR plays a crucial role in amplifying the Raman signal in SERS, making it a valuable analytical tool with extended LOD. However, while SERS demonstrates excellent sensitivity, its repeatability in assays often lags behind that of SPR. Recognizing the strengths and weaknesses of both SPR and SERS, an optimal approach emerges in harnessing the benefits of both techniques through a dual-mode sensor. Such a sensor could facilitate the simultaneous observation of molecular binding events, kinetics and concentration changes. Thus, empowering researchers to unravel intricate mechanisms in biological, chemical, and environmental studies [125–130]. Additionally, combined SPR/SERS dual-mode can provide more options for detection and verify the results to improve the accuracy and reliability of assays. For example, a SPR/SERS dual-mode plasmonic biosensor based on catalytic hairpin assembly (CHA)-induced gold nanoparticle network was proposed for the detection of cancer-related miRNA-652 in serum [125]. The biosensor included capture DNA-functionalised gold nanoparticle (probe 1), H1 and 4-mercaptobenzoic acid (4-MBA) co-modified gold nanoparticle (probe 2), and fuel strand 6-carboxy-X-rhodamine (ROX)-labelled H2. In the presence of miRNA-652, target-triggered CHA reactions were executed to induce the formation of probe 1-probe 2 networked structures, which resulted in the colour changes of dark field microscopy (DFM) images and the enhancement of the SERS signal. The SPR sensing was achieved by extracting the integral optical density of dark-field colour in the DFM images. The SERS sensing was realised by the ratiometric SERS signals of ROX molecules and the internal standard 4-MBA molecule. Both SPR and SERS assays showed linear calibration curves and LODs of 42.5 fM and 2.91 fM, respectively, with % recovery ranged between 94.67 and 111.4%.

In another recent example, Cao et al*.* reported a SPR/SERS dual mode biosensor for the detection of human immunoglobulin (H-IgG) using gold nanogratings [131]. The gold nanogratings were fabricated on a single-crystal silicon surface using wet etching-assisted nanosecond direct laser interference patterning (DLIP), enabling a precise control over period and depth. By optimizing the DLIP intersection angle and etching time, the resulting gold nanogratings exhibited remarkable SPR absorption dips in reflection spectra and robust localised SPR, rendering them highly responsive to local refractive index changes and conducive to strong localised electric fields for enhanced SERS activity. In the SPR mode, protein A immobilization on the gold nanograting surface enabled specific H-IgG binding, achieving a LOD of 23.5 nM. In the SERS mode, the biosensor demonstrated a better sensitivity with a LOD of 1.176 nM. The SPR mode demonstrated a high linearity, while the SERS method enabled sample identification based on characteristic peaks, thus enhancing specificity and accuracy. The synergy between these complementary modes offers multiple detection options and enhances result cross-validation, thereby improving assay accuracy and reliability [131].

In another application, Zhang et al*.* proposed a bimetallic waveguide-coupled surface plasmon resonance (WCSPR) configuration to enhance the Raman scattering of 4-mercaptopyridine with an evanescent field excited using surface plasmons [132]. Incident angle-dependent SERS spectra were measured in the evanescent field on this WCSPR configuration using an in-house-built angle-dependent SPR-SERS micro spectrometer. The SERS signal obtained under evanescent field excitation at the SPR angle was 20 × higher than that collected using the conventional SPR configuration. The experimental results also proved that the waveguide-coupled surface plasmons in this evanescent field-enhanced SERS spectroscopy setup had electric field penetration depth of at least 500 nm, which is longer than the penetration depth for conventional surface plasmons. The SERS enhancement factor of this WCSPR configuration was 6.2 × 10^7^ and the LOD reached 1.0 × 10^–10^ M.

#### Fluorescence spectroscopy

Fluorescence spectroscopy has long been recognised as a valuable detection technique, particularly in biology-related applications. Despite its advantages in rapid imaging and sensitivity, fluorescence suffers from poor multiplexing capabilities due to broad emission features that result in signal overlap. Moreover, organic fluorophores exhibit fluorescence intensity reliant on their chemical environment. Coupled with susceptibility to signal photobleaching, this can profoundly impact data quality. Emerging fluorescent probes like quantum dots (QDs) offered narrower bandwidths and enhanced photochemical stability but present challenges such as surface modification complexity and restricted multiplexing capabilities [133]. SERS stands out as a potent tool for multiplexing, ultrasensitive and quantitative detection. However, it remains a low-throughput imaging method necessitating lengthy acquisition times and delivering lower spatial resolution, particularly for imaging expansive areas, when compared to fluorescence [133]. Therefore, combining SERS and fluorescence measurements in a single platform presents a versatile tool for molecular detection and analysis. In this dual-mode sensing approach, fluorescence is initially monitored for rapid screening, followed by selective SERS measurements at identified areas of interest traced through fluorescence. Accordingly, this approach enables accurate ultra-sensitive multiplex quantification with a high resolution [133–137]. The combination of SERS and fluorescence has been used for the simultaneous imaging and absolute quantification of significantly different expression levels of miRNAs in living cells for the early diagnosis of cancer [138]. This was carried out using a fluorescence-SERS switching nanoprobe, termed as dual-signal switchable (DSS) probe, designed by Ye et al*.* In this design, a target strand displacement reaction was introduced in the probe to amplify the signal n times, termed as 1:n asymmetric signal amplification, that respond only to the low-abundance miRNA. The fluorescence measurement provided a rapid and direct visualization for intracellular monitoring, while a ratiometric SERS detection offered a sensitive quantification with narrow characteristic peaks for multiplex qualitative evaluation of miR-21 and miR-203 [138]. The peak ratiometric SERS intensities were used to quantify miR-21 and miR-203 in the range 10^–13^–10^–7^ M and 10^–15^–10^–9^ M, respectively.

**Fig. 6:**
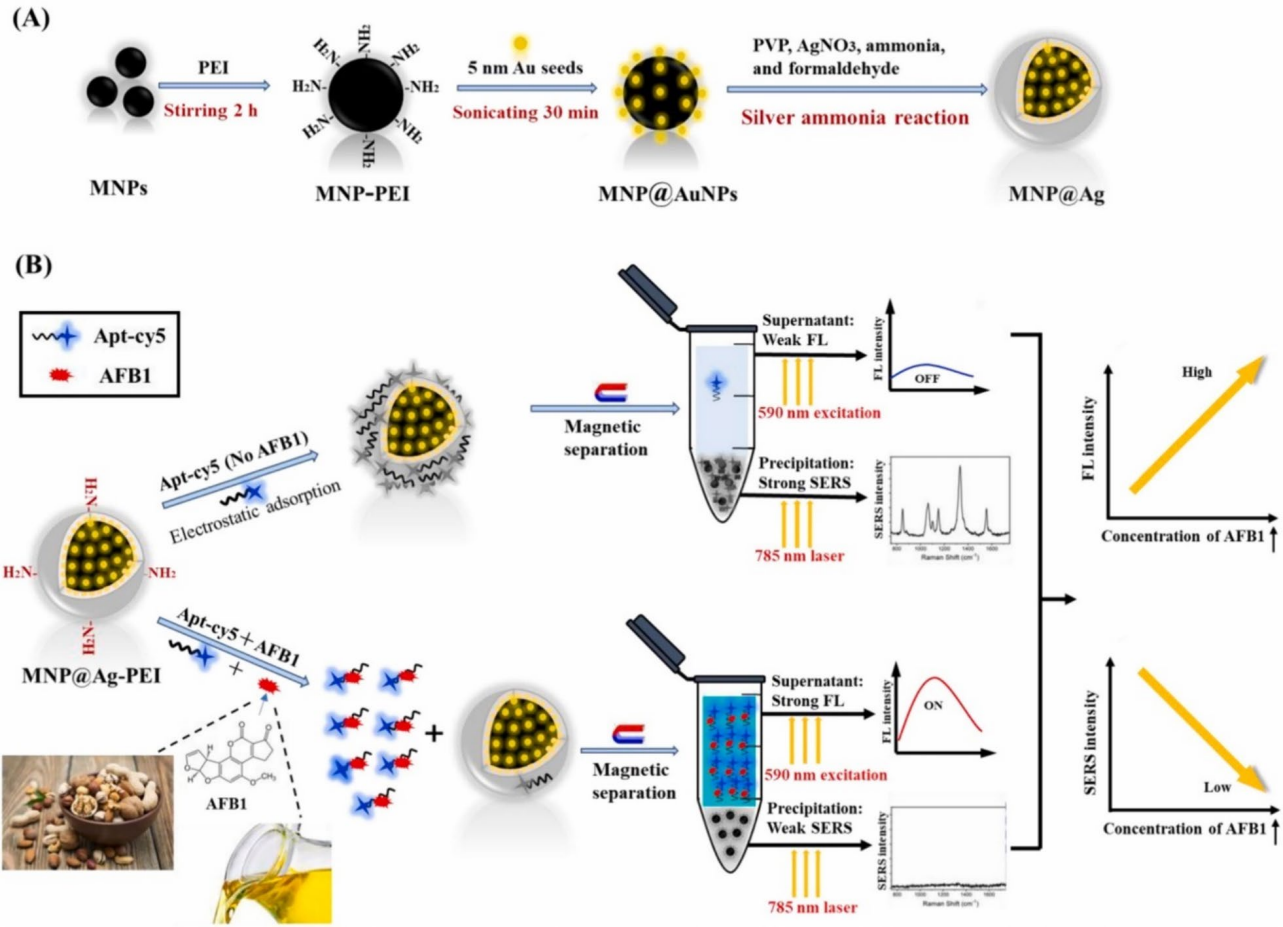
**A** Schematic illustration of the fabrication of MNP@Ag using core MNP coated in PEI, followed by the addition of 5 nm gold seeds. The continuous silver shell is then formed through a silver ammonia reaction. **B** Schematic showing the principle of a SERS-fluorescence dual-signal aptasensor for AFB1 determination. When only Apt-cy5 is added to the MNP@Ag-PEI, it electrostatically absorbs onto the surface. When magnetic separation takes place, a strong SERS and weak fluorescence signal is observed. If Apt-cy5 and AFB1 are added together, they specifically bind, preventing absorption to the MNP@Ag-PEI. After magnetic separation, a strong fluorescence and weak SERS signal is obtained. Reprinted from ref [139], copyright 2023, with permission from Elsevier

Another dual SERS-fluorescence aptasensor design was described by He et al*.* for the quantification of aflatoxin B1 (AFB1) contaminated nut samples, using fluorescent dye cyanine5 (cy5) as a fluorophore and Raman reporter [139]. Polyethyleneimine modified silver coating magnetic nanoparticles (MNP@Ag-PEI) were prepared and used to absorb a cy5 modified aptamer (apt-cy5), which have a specific binding affinity towards AFB1. In the absence of the toxin, the apt-cy5 was absorbed on the surface of MNP@Ag-PEI. Thus, the SERS intensity of cy5 increased. After magnetic separation, the precipitation exhibited a strong SERS intensity, while the supernatant showed a weak fluorescence signal. Conversely, in the presence of the toxin, the specific binding between AFB1 and apt-cy5 prevented the absorption of apt-cy5 on MNP@Ag-PEI surface. Accordingly, after magnetic separation, the SERS intensity of precipitation was weakened, and the supernatant showed a high fluorescence intensity (Fig. 6). Therefore, the aptasensor was used to quantitatively determine AFB1 in nut samples via the decrease in SERS signal and the increase in fluorescence intensity. The results indicated linear quantification in the ranges of 0.001–1000 ng/mL and 0.2–20,000 ng/mL, with detection limits of 0.45 pg/mL and 135 pg/mL for the SERS and fluorescence methods, respectively. In another combined approach, Zou et al*.* reported a dual SERS-fluorescence immunoassay to detect tuberculosis antigen CFP-10, using graphene quantum dot (GQD) labels and a sensing platform of linearly aligned magneto plasmonic nanowires (MagPlas NWs) [140]. GQDs were used as bi-labelling materials for the simultaneous SERS and photoluminescence detection. CFP-10 antigen was detected using two different antibodies (G2 and G3) following a sandwich-type immunoassay, where G3 functionalised GQDs were bound to G2-functionalised MagPlas NWs in the presence of the complementary target (CFP-10). With this sandwich-type immunoassay using dual-mode nanoprobes, both SERS signals and fluorescence images were recorded in a highly sensitive and selective manner with a LOD of 0.0511 pg/mL.

Overall, a single analysis technique is typically insufficient for the accurate quantification of a target analyte. Each technique has its own advantages and drawbacks. Correlation of more than one analytical technique in a single platform provides the potential to realise the complementation of the individual advantages and to remedy the deficiencies of each technique, as well as enabling more precise and multi-dimensional characterisations for the analytes. SERS correlative techniques have markedly improved the quantitative detection and characterisation capabilities of SERS. Such correlations have also enhanced the capacity to determine multiple analytes within complex matrices.

## Quantification in SERS-based imaging

By scanning the sample with a focused laser beam and collecting the enhanced Raman signals at each point, a spatially resolved image based on the vibrational spectra can be obtained without damaging the sample. The ability to acquire large quantities of spectra lends itself to analysing and characterising surfaces, including analyte detection based on interactions that occur at the surface of the substrate. Beyond analysing substrates, quantitative SERS imaging has been applied for the detection of influenza virus A, to understand mesenchymal stromal cell differentiation and monitor intracellular dynamic of live cells [141–148]. As was discussed, combining techniques aids in validating the proposed quantitative analysis of SERS-based imaging. For example, a core-satellite gold nanostructure was employed for the in vivo quantitative detection of H_2_O_2_ produced during inflammation and cancer in mice and rabbits [149]. The SERS imaging enabled the accurate differentiation between the inflamed region and normal tissue. Photoacoustic imaging was used in parallel with SERS-imaging to validate the determined H_2_O_2_ concentrations.

Although there are examples demonstrating the quantitative detection of analytes by SERS-based imaging, it is still traditionally used in a qualitative manner. Here, we use the term nanotag to describe a nanoparticle that is functionalised with a Raman reporter, encapsulated in a protective shell, and is equipped with a moiety capable of targeting an analyte of interest. The overall design of SERS nanotags allow for compatibility with a wider range of applications beyond just detecting analytes in solution. Of note is the use of these nanotags for imaging, particularly bioimaging [43, 150–160]. Reminiscent of immunohistochemistry, different flavours of nanotags can be used to simultaneously detect the presence of multiple biomarkers. Furthermore, this can be applied to differentiate between cell types present within the same sample. For example, differentiating between U251 (malignant glioblastoma, model for mesenchymal phenotype), LNCaP (human prostate adenocarcinoma, model for epithelial phenotype), and PBMC (peripheral blood mononuclear cells) cells was achieved using three flavours of nanotags [161]. The first nanotag had a combination of anti-CD44 and anti-NCad antibodies to target mesenchymal cells, the second had a combination of anti-EpCAM and anti-E-Cad antibodies to target epithelial cells, and the third had anti-CD45 antibodies meant to target CD45, which is broadly expressed on white blood cells. The authors observed that the combination of these three nanotags when processed using a random forest algorithm allowed for sufficient differentiation of the various cell types within a sample mixture as the second nanotag only interacted with the LNCaP cells, while the other two nanotags did interact with both the U251 and PBMC cells, but in different ratios. By generating larger quantities of distinct nanotags, it becomes possible to differentiate increasing numbers of cell types [162].

For SERS-based imaging experiments, it is often necessary to incubate large numbers of nanoparticles with respect to the number of cells present, to generate an observable response. In a recent work, Bagheri et al*.* found that a ratio of 18,000 nanotags per cell resulted in the highest ratio of specific to non-specific binding of nanotags to A431 cells [163]. The authors noted that this value was determined based on the observation that when the concentration of epidermal growth factor receptor (EGFR) nanotags was lower, a weaker SERS signal was observed due to fewer interactions between the nanotags and the receptors. When higher concentrations of nanotags were used, the IgG nanotags that were present in the multiplex measurements to act as a control had greater non-specific binding to other receptors present at the cell surface. This observation raises an important point, which is that the number of nanotags needed per cell will naturally depend on the cellular target. Prior analysis of A431 cells estimated 5 × 10^5^ EGFR receptors per cell (assuming a cell diameter of 25 µm) [164]. This would yield an estimated receptor to nanotag ratio of 28:1. More importantly though, it is unknown how many nanotags are present per cell. This question represents a predominant challenge in the traditionally qualitative analysis of SERS-imaging. Furthermore, even in analyte detection, knowing the effectiveness of nanoparticle uptake is critical because if the uptake is poor, the capability for quantitative analysis will be severely hindered.

Transitioning to tumour and animal models, the multiplexing capabilities of SERS-based imaging not only enables the analysis of multiple tags simultaneously [162], but it also allows for monitoring processes including uptake of nanoparticles into tumours [165]. This can be extended to comparing different sizes of nanoparticles to ascertain the ideal dimensions for uptake in tumours, and how that can compare with other biological systems [158]. As was discussed throughout the previous sections, transitioning from qualitative to quantitative detection requires a metric by which a calibration curve can be generated. Arguably the simplest method would be to consider the SERS signal that originates from a single nanoparticle. This, of course, represents a series of challenges as the SERS signal originates from just a fraction of the total surface area [166], variation in size and shape leading to changes in the electromagnetic enhancement, polarization dependence, fluctuations in the signal as a result of chemical transformations of the Raman reporter [167], etc. With these limitations in mind, the following sections explore current approaches that have been used to ascertain the number of nanotags (or nanoparticles) present within a cell using SERS-based imaging.

### Correlative SERS-mapping and electron microscopy

**Fig. 7:**
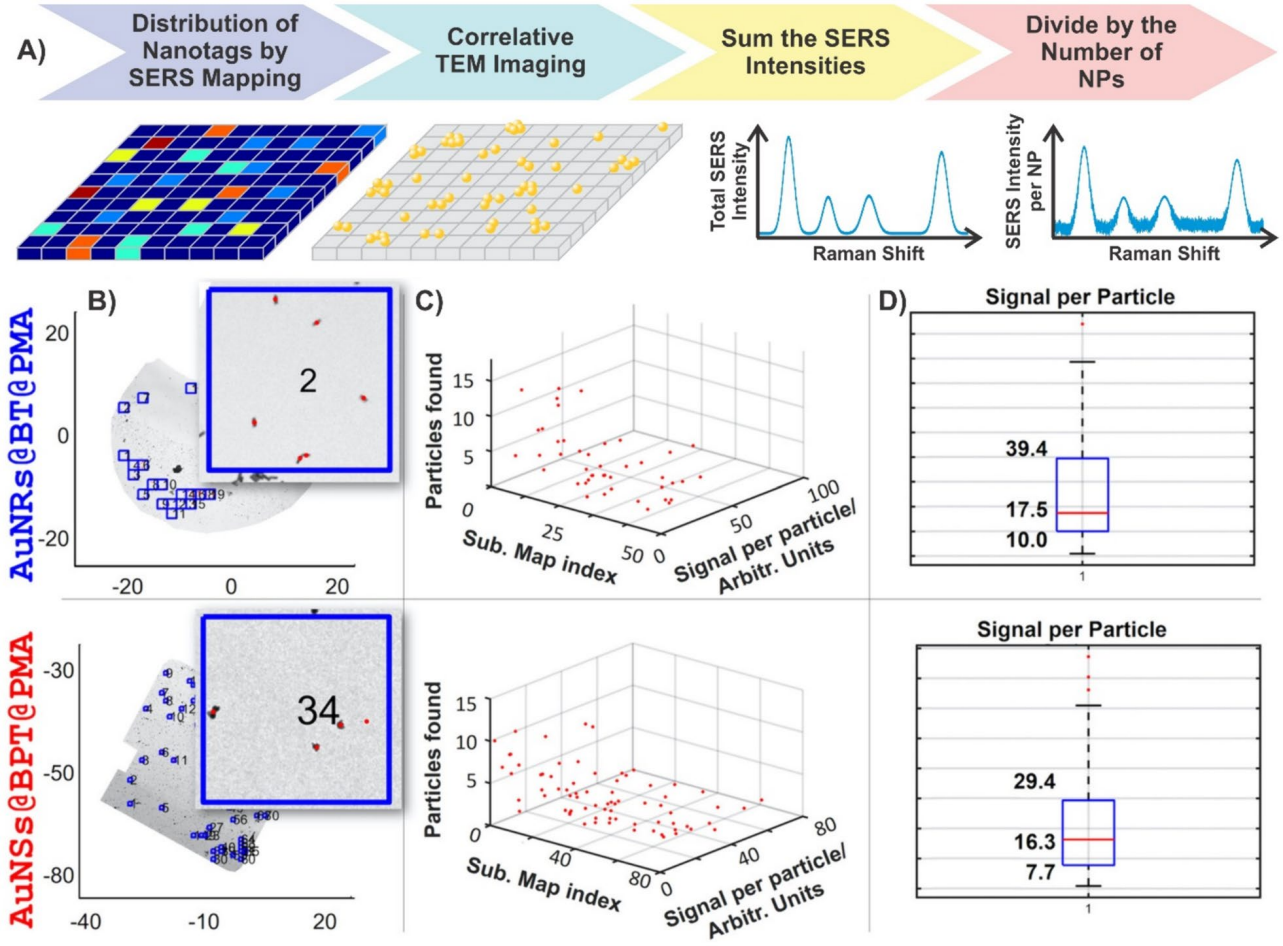
**A** Workflow diagram detailing a method for determining the average SERS intensity per nanotag using a combination of SERS mapping and correlative TEM imaging. **B**–**D** Experimental results using this approach and the SERSTEM app. [174] Copyright 2020 Wiley. Used with permission from ref.

The most straightforward approach to determine the number of nanotags present within an area is to simply count them. This can be achieved by taking TEM images of cells incubated with the nanotags. This process is, however, extremely arduous, time-consuming, and not cost effective. As such, developing an alternative methodology that can still benefit from the high-resolution capabilities of electron microscopy is of value. The combination of electron microscopies (transmission or scanning) has been applied in various ways to understand how the geometry of nanoparticles and the presence of multimers can influence the SERS response [168–172]. It was proposed that for pegylated gold nanoparticles functionalised with a Raman reporter, it may be possible to ascertain the number of nanoparticles present based on the polynomial relationship observed between SERS intensity and the number of nanoparticles present [173]. Figure 7A describes a general workflow for performing these types of measurements. After the sample has been deposited onto the appropriate substrate, a SERS map is taken, whereby, the false colour image provides insight into the relative distribution of the SERS intensities. The sample is then imaged using the corresponding electron microscopy, such as TEM. Here, the presence of identifiable markers in the sample allows for the two images to be correlated. In the case of TEM grids, the presence of labelled grids simplifies this. By correlating the SERS map with the TEM image, it is possible to determine the number of nanotags present within each pixel of the SERS map. From here, the data processing can differ, with the workflow following the protocol of Lenzi et al*.* [174]. As opposed to dividing the intensity within each pixel by the number of nanotags present to determine the SERS intensity per nanotag, the SERS intensities from all pixels were summed and divided by the total number of nanotags. This methodology for processing the spectra was packaged as an app called SERSTEM, with the results for gold nanorods functionalised with benzene thiol, and gold nanostars functionalised with biphenyl-4-thiol shown in Fig. 7B–D. The plots of the signal per particle highlight an important aspect when exploring multiplexing capabilities, and that is the variation in the SERS intensity between flavours of nanotags or using different types of plasmonic structures. For example, the SERS map associated with the distribution of one flavour of nanotag may have a higher SERS intensity, simply because that nanotag is intrinsically brighter than its counterpart(s). As such, a false sense of the number of nanotags present may be reached. Careful preparation of the nanotags such that they have comparable SERS intensities, or, characterisation of the SERS response per particle is therefore critical.

### Correlative SERS-mapping and mass spectrometry

With the correct calibration method, mass spectrometry can be used for quantitative analysis. ICP-MS has shown great promise for quantifying metal content in biological samples post incubation with nanoparticles [175, 176]. In a typical approach, the biological sample is digested in aqua regia, and the metal content (i.e. gold) is determined having developed a calibration curve using gold standards or the nanoparticles themselves. The absolute metal content can then be determined allowing for various metrics to be compared, such as how the relative size of the nanoparticle influences uptake, or, where nanotags and nanoparticles distribute within the various organs in a body. Given the strength of ICP-MS as an analytical technique, combining it with SERS could therefore provide a metric that the SERS signals could be calibrated against.

**Fig. 8:**
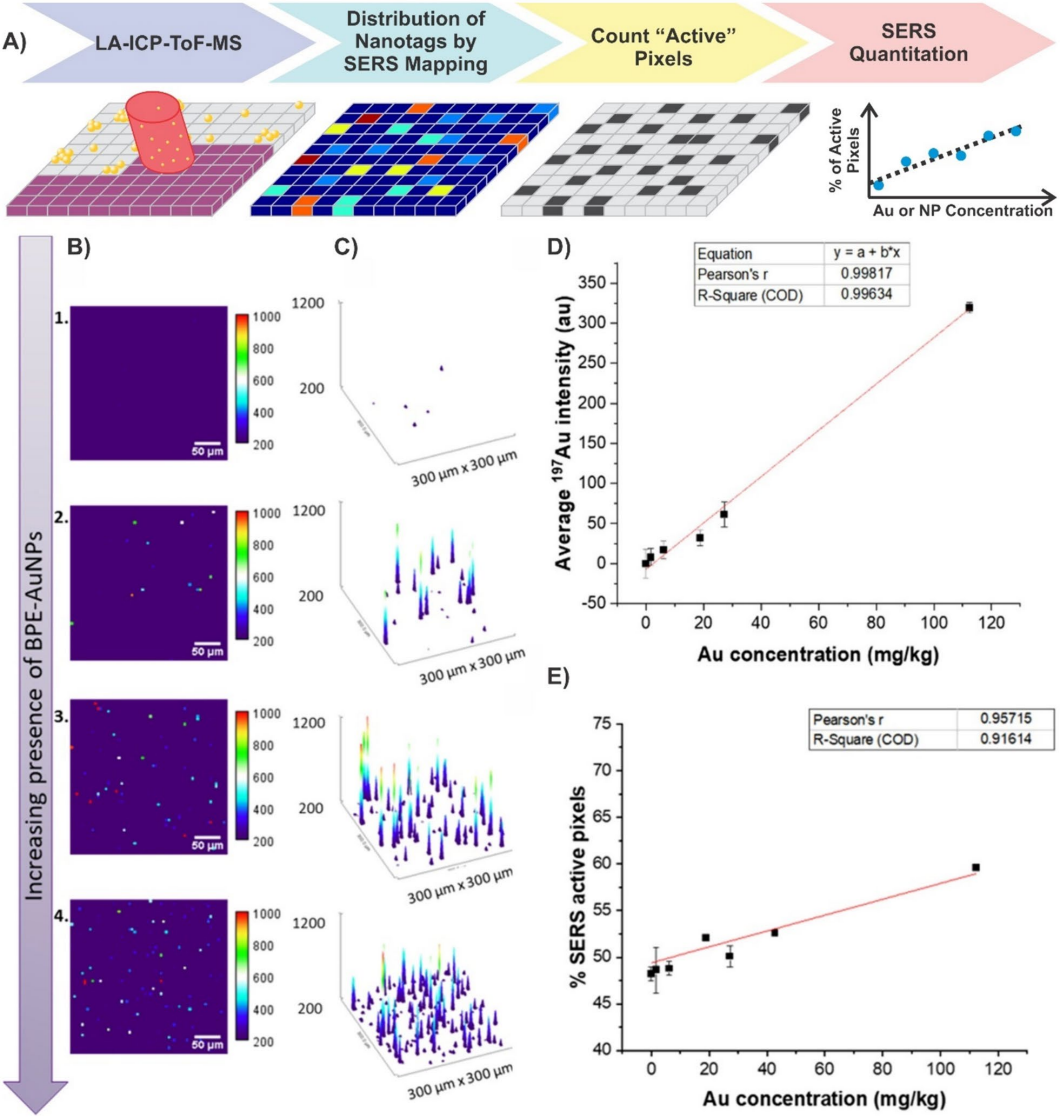
**A** Workflow diagram detailing a method for quantifying the SERS signal in a map using the number of active pixels and the gold concentration. **B** False colour images of the absolute intensity in SERS maps obtained of different standards. **C** Surface plots illustrating the number of SERS events, corresponding to the false colour images depicted in **B**. The height of the spikes is relevant to the colour scale shown in **B**, while the number of spikes increases with increasing amounts of BPE-gold nanoparticles and therefore “active pixels”. **D** Characterisation of calibration standards by LA-ICP-ToF–MS. Error bars represent ± RSD of intensity (n = 3 replicates). **E** Calibration curve showing a linear increase between the percentage of SERS active area and the Au concentration present in the standards. Error bars correspond to mean ± SD (n = 2 replicates per condition with 3600 spectra per replicate). Adapted with permission from ref. [177]. Copyright 2023 The Authors

The most notable example is the work of Leventi et al*.* [177], with a workflow diagram provided in Fig. 8A. To better replicate a potential biological environment, such as tissue, the authors developed an approach whereby functionalised gold nanoparticles were incorporated into a gelatin matrix. A 3D printer was used to produce gelatin droplets that contained different amounts of gold nanoparticles. After allowing the droplets to dehydrate, thin gelatin sections with dispersed gold nanoparticles were formed. Laser ablation inductively coupled plasma time-of-flight mass spectrometry (LA-ICP-ToF-MS) was then used to validate the distribution of the gold nanoparticles throughout the gelatin standards. As was the case for the correlative electron microscopy approach, SERS mapping was performed over regions of the gelatin sections. Instead of relying on the intensities of the SERS signals within the pixels of the map, the authors used an “active-area” approach, where pixels were defined as being “active” or “inactive.” Comparisons between how best to define these pixels found that setting a minimum threshold intensity for the main vibrational mode of the Raman reporter was most effective. A linear relationship was subsequently found for the percentage of active pixels within the total map compared to the total amount of gold present within the gelatin standards. Using single particle ICP-MS (spICP-MS) of the gold nanoparticles, it is possible to convert the total mass of gold present within the standards to the number of gold nanoparticles present. Figure 8B–E highlight the experimental results that yielded the calibration model from the SERS mapping (Fig. 8B and C), ICP-MS-ToF analysis of the standards (Fig. 8D), and finally the SERS quantification calibration curve (Fig. 8E). This calibration model provides an effective method of nanoparticle quantification and demonstrates the potential of quantitative SERS imaging, which could be applicable to biological models, such as cells or tumours, where the nanoparticle concentration could be correlated with biological responses.

### Quantification in 3-dimensional SERS imaging

The two previously described methods focussed exclusively on 2-dimensional imaging. However, for many applications, such as the incorporation of nanoparticles and nanotags into biological samples, such as cells, 3-dimensional imaging is required [178]. First and foremost, only by analysing the entirety of the cell is it possible to know the total number of nanoparticles present. More importantly though, is the ability to ascertain their distribution within the sample, such as localization within cellular region(s). The former can be determined using conventional methods, including ICP-MS, however, it is a destructive technique. Imaging the sample by TEM can address the latter, but once again, it is an inefficient process requiring additional sample preparation. These techniques do however provide a means of validating the newly developed SERS-based approaches [151, 179].

To evaluate the content of gold nanoparticles in cells during mitosis [151], Lenzi et al*.* applied their earlier developed approach discussed in Sect. 4.1 [174]. Although the approach did show promise as it generally agreed well with the correlated ICP-MS results [151], it was found that at the three longest time points, corresponding to the greatest number of cell divisions, and thus the fewest number of gold nanoparticles per cell, the differences became greater. The discrepancy was attributed to the weaker SERS signal of the gold nanoparticles. To overcome this, the authors transitioned from a supervised method to an unsupervised method. As opposed to summing the SERS intensities and dividing by the estimated intensity of a single nanoparticle, a non-negative matrix factorization (NMF) approach was applied as they can be used to recover the SERS profile of the analyte without the need for a reference spectrum, even in the presence of spectral interference [180, 181]. Besides the unsupervised nature of this approach, the important aspect used for quantification was the number of spectra (events) that had a Pearson’s linear correlation coefficient of ρ ≥ 0.75 with respect to the SERS spectrum found during the NMF method. Compared to the supervised approach (SA), the digital unsupervised algorithm (DUA) had better agreement with the number of nanoparticles present per cell estimated by ICP-MS when fewer nanoparticles were found. It is worth noting that at higher nanoparticle concentrations, the probability of more than one nanoparticle being present within an active pixel increases. Under these conditions, the DUA may underestimate the number of nanoparticles present compared to the SA. Thus, applying both supervised and unsupervised approaches can be of benefit when the number of nanoparticles present is unknown, as both are approaches are effective over different ranges.

**Fig. 9:**
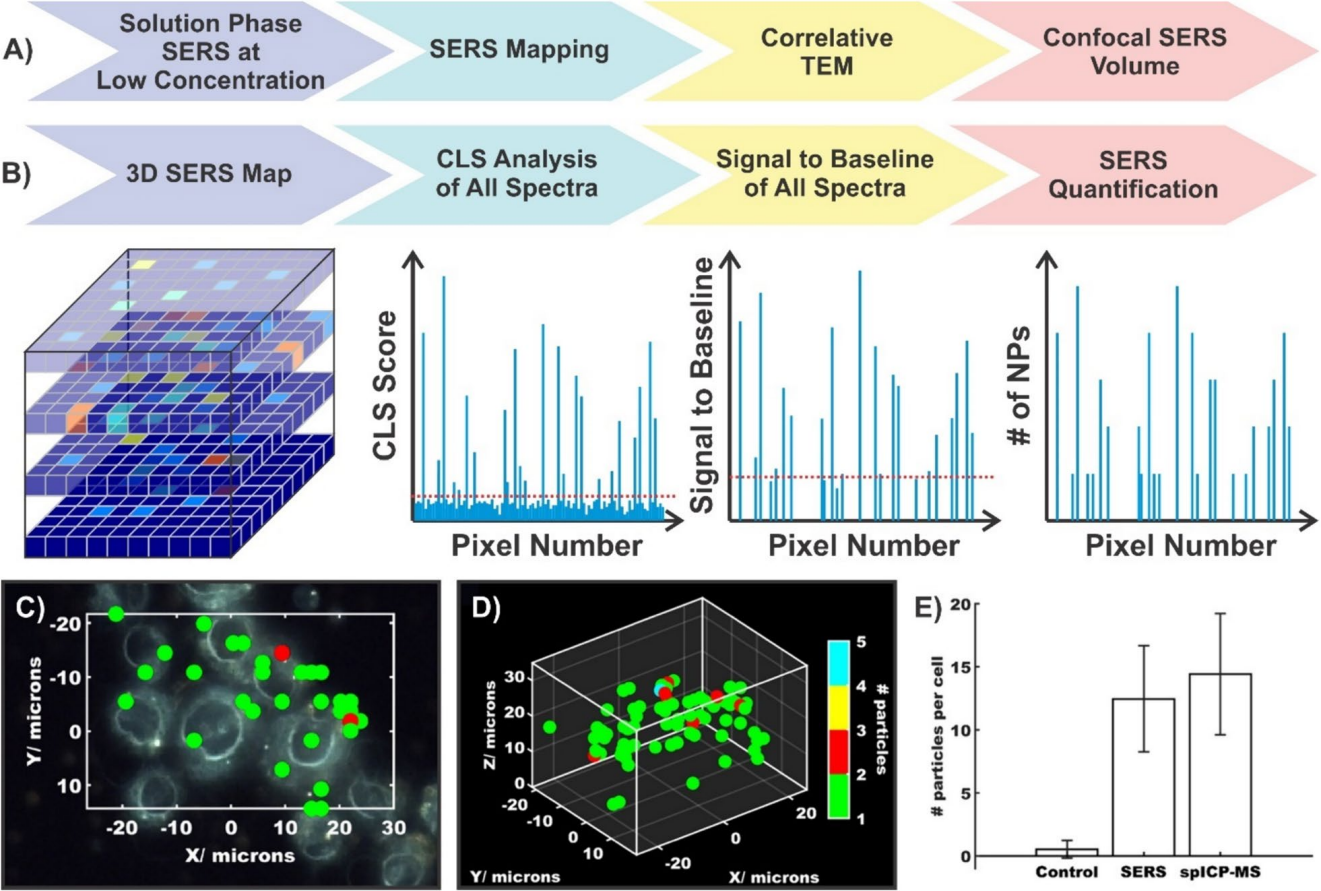
**A** Workflow diagram for the characterisation of gap-enhanced Raman tags (GERTS) that were subsequently used in 3D mapping experiments. **B** Workflow diagram for the quantifying the number of nanoparticles present within a cell by determining the signal to baseline of a single nanoparticle using the classical-least squares scores from the spectra within the 3D map. Experimental results highlighting the distribution of GERTs in **C** a single *z*-plane, and **D** across the full 3D map where multiple cells are present. **E** Average number of nanoparticles per cell determined by SERS and sp-ICP-MS. Adapted with permission from ref. [179]. Copyright (2024) American Chemical Society

More recently, Scarpitti et al*.* introduced an alternative methodology for quantifying the number of plasmonic nanoparticles in cells [179]. A critical portion of that work was the thorough analysis of different types of plasmonic nanostructures, to understand their behaviours at or near the single-particle level. Of the evaluated structures, gap-enhanced Raman tags (GERTs) were found to be ideal. Figure 9A highlights some of the characterisation methods that were used, including both solution phase and on a substrate. Evaluating the confocal volume for the mapping experiments, especially in the *z*-axis was an important step often overlooked in measurements. By knowing the confocal volume of the laser spot, it became possible to select appropriate *x*-, *y*-, and *z*-step-sizes during the 3D SERS mapping experiments such that the contributions from outside of the laser spot were minimised.

Similar to the previous example [151], processing of the spectra acquired from within the map was used to ascertain the number of nanoparticles present within each pixel (Fig. 9B) [179]. After acquiring the map, classical least-squares analysis was performed on the spectra, and those spectra that met a defined threshold were further analysed. The signal to baseline of those spectra were plotted, where a new threshold was determined, corresponding to the signal to baseline intensity of a single nanoparticle. The output of such an analysis on a series of cells is shown in Fig. 9C–E. By comparing the spatial coordinates of each pixel with the positions of the cells, and the signal to baseline intensity within those pixels, it was possible to determine the estimated number of nanoparticles present per cell. As Fig. 9E shows, the results of this analysis compared very well to that of the ICP-MS results (12 ± 4 vs. 14 ± 5 particles per cell).

Although quantification of the number of nanoparticles within a cell by SERS is still in its infancy, both examples highlight that careful analysis of the data is required. Thorough characterisation of the nanoparticles is critical, as is evaluating different approaches to interpreting the data. Given the limited current literature, benchmarking new methods with established ones will be of great importance. Likewise, validating the values determined by SERS by alternative quantitative techniques, such as TEM and ICP-MS, continues to be an essential step. Furthermore, expanding the number of cells analysed, and thus the amount of data acquired, is also important to ensure that under sampling is minimised, and that the results obtained are truly a reflection of the nanoparticles present within a 3D volume.

### Anticipated challenges moving forwards in quantifying nanoparticle content

Given the desire to expand into multiplexing for imaging [162, 163, 182], the strategies to determine the SERS intensity per nanotag will still be applicable, however the methods for validating the number of nanotags per cell must be considered. As was mentioned in the previous section, TEM imaging and ICP-MS remain the methods of choice for validating the number of nanotags present within the biological sample. However, given the classical design of nanotags that have a gold nanoparticle core functionalised with an organic Raman reporter, both techniques will likely struggle to differentiate between flavours of nanotags. Correlative SERS mapping and TEM imaging may be possible, but will be a slow and tedious process with each individual pixel being analysed. If both nanotags are present within the same pixel of the Raman map, the issue of differentiation once again occurs. If the only metal present is gold, it will not be possible to distinguish between the different flavours of nanotags present by ICP-MS. In this regard, the next generation of nanotags may need to integrate alternative materials and Raman reporters.

Since the interactions between nanoparticles and cells are both size and shape dependent, it is important to optimally compare the effectiveness of different nanotags, that they have similar physical properties. It may not be possible to prepare similarly sized and shaped nanotags if additional material layers are added. In this regard, maintaining a consistent inner gold nanoparticle may be advantageous. As a result, it would be necessary to rely on the Raman reporter to differentiate between flavours. Traditional Raman reporters, namely organic dyes, pyridine derivatives and aromatic thiols, are not suitable for this purpose. Instead, alternative metal containing compounds are needed. Transition metal carbonyls offer potential benefits in this endeavour [183]. The presence of different metal ions (molybdenum, ruthenium, tungsten, rhenium, osmium) with the M(CO)_x_ Raman reporter would allow for SERS-based multiplexing as depending on the nature of the molecule, unique vibrational modes are present in the cell silent region between 1900 and 2200 cm^−1^ [184]. Also, more importantly, this would allow for distinct metals to be detected by ICP-MS. To the best of our knowledge, that aspect has not been explored, but the applicability of using the M(CO)x vibrational mode in cell imaging has. Using a triosmium carbonyl cluster Os_3_(μ-H)_2_(CO)_10_ as the Raman reporter [185], Kong et al*.* demonstrated uptake of the nanotags containing a transition metal carbonyl through SERS mapping of oral squamous cell carcinoma cells. Successful targeting was then shown by incubating ovarian carcinoma cells with the same nanotags. As these cells did not contain the target epidermal growth factor receptor, no SERS signal for the triosmium carbonyl cluster was observed. Alternatively, more conventional Raman reporter designs may be adapted [186]. Here, the synthesised Raman reporter was reacted with cisplatin to create a new means of drug delivery, though this type of approach could potentially be adapted. The important aspect was that much like the M(CO)_x_, the presence of the dialkyne allowed for a vibrational signal to be detected within the cell silent region. Furthermore, correlative detection of both metals by ICP-MS allowed for validating the presence of the cisplatin on the gold nanoparticles once the conjugate system entered the cells. This demonstrates the concept of using a secondary metal to further confirm the presence of the nanostructure.

## Quantification at depth

As was established in the previous section, SERS-based mapping experiments are predominantly focussed on the number of nanoparticles present within the imaged area. With the extension to 3-dimensional imaging, it may become possible to have insights into where the nanoparticles are located in a general sense within the sample. However, as the size of the sample increases, specifically the thickness, SERS-based imaging often becomes insufficient as spectral interference from the sample, such as absorption and scattering, becomes more prevalent. Spatially offset Raman scattering (SORS), and its plasmonic alternative, surface-enhanced SORS (SESORS), have emerged as attractive techniques for detection through barriers [187–192]. Details regarding the specifics of these techniques are outside the scope of this review and have been more thoroughly described in other reviews [193, 194].

In terms of quantitative analysis, spatially offset (or transmission) Raman typically emphasises two areas: (i) the maximum depth through which detection can occur, and (ii) the spatial position of a target of interest. The former is a relatively straightforward process. The thickness of a barrier, commonly some form of tissue, is gradually increased with the spatial offset subsequently increased. Once the target spectrum can no longer be discerned, either by visual inspection or through data analysis (i.e. principal component analysis), the maximum depth can then be determined. The development of brighter nanotags in combination with alternative collection geometries has allowed for detection to progress from a few millimetres to several centimetres [195–199]. On the other hand, the latter presents a greater challenge.

Unlike confocal Raman measurements where the beam diameter is often on the order of 1 µm, depending on the set-up used, the beam diameter for SORS measurements will typically be in the 100’s of µm to a few mm’s. As such, the focus transitions away from analysing small biological samples (i.e. cells) to larger materials, such as tumours. Given that the detection in (SE)SORS occurs through a barrier, quantification in imaging includes not just the depth of the target, but its spatial coordinates. In this regard, prospects of this type of imaging would include determining the margins of a tumour without the need for exposing it during surgery. As such, effort has been made in developing experimental approaches so that the necessary mapping can occur [200–206], including the use of custom set-ups, as well as the incorporation of commercial instruments with *x*,*y*-translation stages.

**Fig. 10:**
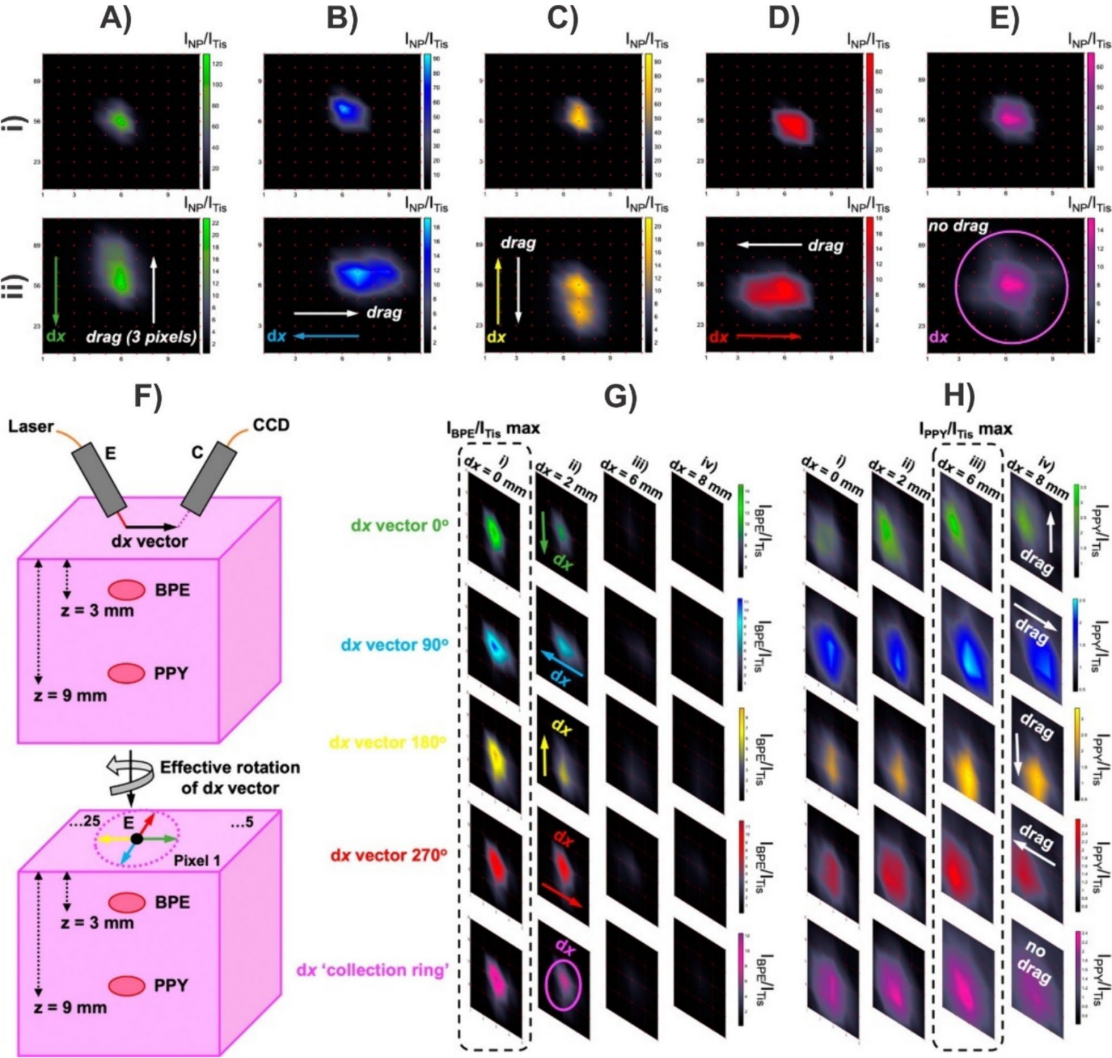
Ratiometric images of plasmonic powder buried 3 mm beneath tissue where the images were acquired after having rotated the sample **A** 0°, **B** 90°, **C** 180°, and **D** 270°. **E** Average ratiometric image across the four linear offset vectors to mimic a ring-collection offset. Images were acquired at linear offset magnitudes of (i) 0 mm, and (ii) 8 mm. **F** Schematic representation showing the spatial discrimination experimental set-up where two flavours of plasmonic powder (BPE, and PPY) are buried at different depths within a tissue sample. Ratiometric images of the **G** BPE plasmonic powder buried 3 mm beneath the surface, and **H** PPY plasmonic powder buried 9 mm beneath the surface. Images were acquired with linear offset magnitudes of 0, 2, 6, and 8 mm. For each linear offset, ratiometric images were collected after having rotated the sample from 0 to 360° in 90° increments, with the average image reflecting a ring-collection offset. Adapted with permission from ref. [44]. Copyright 2022 The Authors

However, in order for these approaches to be clinically relevant, it is necessary for the image generated to be an accurate reflection of the true position (*x*,*y*,*z*) of the target of interest. The work of Berry et al*.* noted an important observation for SESORS images when taken using linear offsets [44]. As can be seen in Fig. 10A, when the SESORS image is generated for a linear offset, as is needed for depth measurements (i and ii), the ratiometric image shows a blurring effect. The authors described this as image induced drag. The position of this drag changed as the sample rotated with respect to the position of the optical set-up (Fig. 10A–D). By averaging the four images together, it becomes possible to create an image that minimises the image induced drag (Fig. 10E) and reflects a ring-collection approach [207]. To validate that such an approach is viable for detection at different depths, plasmonic powders made of gold nanoparticles functionalised with one of two Raman reporters (1,2-bis(4-pyridyl)ethylene (BPE), or 4-(1*H*-pyrazol-4-yl)pyridine (PPY)) were positioned at two different depths (3 mm for BPE, 9 mm for PPY) as shown in Fig. 10F. The corresponding ratiometric images (Fig. 10G and H) show the characteristic image induced drag until the images are averaged. Importantly, by performing the measurements with different linear offsets, it became possible to differentiate the depths at which the plasmonic powders were located. Subsequent work by Xie et al*.* using a transmission Raman scattering configuration [208], developed a two-stage tomographic approach. Using a cylinder of tissue with embedded inclusions of plasmonic nanoparticles, axial measurements, where the excitation and collection optics move along the *z*-axis of the cylinder, were used to determine the depth of the nanoparticles within the tissue. Subsequent ring collection measurements, where the sample was rotated, were then taken at the determined *z*-positions, to ascertain the lateral coordinates of the nanoparticles. It was found that angle increments between 10 and 30° for the ring collection step were sufficient to accurately determine the position of the nanoparticle inclusion (absolute error < 2 mm).

Moving forward, a means of validating the spatial accuracy will be needed. In the highlighted proof-of-concept studies, the positions of the nanoparticle inclusions were defined by the experimenters. This will not be the case in clinical samples. While techniques such as laser ablation inductively-coupled plasma mass spectrometry (LA-ICP-MS) may be applicable for determining the lateral coordinates of the nanoparticles, the need for analysing through large depths of samples could prove challenging. For example, the initial SESORS image could be used to narrow the region of interest, with subsequent cross-sections of the sample taken at that position. The individual sections could then be analysed by the previously described techniques, including LA-ICP-MS. However, such an approach would likely be time-consuming. Alternatively, a second tomographic approach, such as magnetic resonance imaging (MRI), could be explored, as was demonstrated by Nicolson et al*.* [203]. Although not strictly required, hybrid nanostructures compatible with various imaging techniques and SERS have been developed [209, 210], which could improve the image quality. Given that contrast imaging techniques are well established, this would have the benefit of using clinically relevant approaches as benchmarks that the newer method of SESORS imaging can be compared with. By correlating the images, it would then be possible to better establish how accurate the SESORS imaging is, compared to conventional methods.

## Outlook and conclusions

Concurrent with the advancement in SERS, machine learning and artificial intelligence has seen a remarkable increase in popularity and combining these two approaches could prove to be incredibly powerful [211–215]. Qualitatively, these combinations have been used to differentiate origins of extracellular vesicles [89], DNA and RNA [216], bacteria [217], variants of viruses [218], and radiation treatment effects [219]. These methods can be adapted for quantitation [220], though how the final concentration is determined is commonly a ‘black box’. A calibration curve is often shown of predicted concentration with respect to actual concentration [221–225], but understanding how the predicted concentration was found is not necessarily intuitive, especially for a novice. This is not always the case, as calibration curves of the principle component analysis (PCA) score with respect to dye concentration was previously used to demonstrate quantification of food colourants [226]. In this case, the calibration curve is reminiscent to the classical SERS intensity or peak area that most SERS-based calibration curves use. Regardless of the approach and algorithm(s) applied, as has been discussed throughout this review, validating the determined concentration by SERS and machine learning, with a secondary technique, such as mass spectrometry, will help further demonstrate the capabilities of the new SERS-based methods. Not only could qualitative assignment of the origin of the SERS spectrum be identified, but the concentration as well.

There is little doubt that SERS is a powerful technique. But it must be recognised that quantification and reproducibility go hand in hand. For analytical techniques, if the methodology isn’t reproducible, then it will not be possible to generate an appropriate calibration curve, rendering quantification impossible. This reproducibility also extends to other labs being able to replicate the approach used. At its core, this review has addressed three critical questions: (i) can SERS be made quantitative? (ii) if so, how do you validate the proposed quantification? (iii) how do you convince others that SERS is an attractive alternative? Although these questions are distinct, the answers are all linked.

If one believes the literature, then SERS can be quantitative. Given the ability to observe the presence of specific spectra or the change(s) in a spectrum, an appropriate calibration curve can be made. However, as this review has consistently mentioned, the SERS substrates themselves play a vital role in this. In much the same way that if a separation column cannot be made reproducibly, then individual calibration curves will be widely different; the same holds true for the SERS substrates. Given that the contribution of the SERS signal is likely coming from just a small fraction of the surface, being able to prepare the SERS substrates consistently is critical. The same holds true for their stability. Establishing a series of best practices for characterising and comparing them, including a maximum threshold for the relative standard deviation, are important steps moving forward. Furthermore, the incorporation of more interlaboratory studies, that include synthesis, data acquisition and analysis, and calibration curve generation, will also help better establish the quantitative capabilities of SERS.

As was also discussed and highlighted, convincing others that SERS is quantitative requires comparing the obtained results with well-established techniques. These techniques can not only help support the proposed calibration curve, but they also provide a means of direct comparison and results validation. For SERS to become an acceptable method across various fields, it must be benchmarked against the current gold standard techniques being used. Has the limit of detection, limit of quantification, sensitivity, selectivity, specificity, etc. improved? These questions will need to be answered before it is possible to convince others to adopt SERS as an acceptable technique for a desired application, especially when an established protocol is already in place.

Moving forward, for those in the plasmonics and SERS community, helping others to recognise the strengths and capabilities of SERS will become increasingly important for SERS to even be partially adopted let alone widely adopted. It is also necessary to recognise that SERS has limitations, including some that are intrinsic. Understanding this can potentially help narrow the field of focus. Regardless, with more development, both in fundamental areas and applications, along with interlaboratory cooperation, hopefully many of these limitations will be identified and overcome such that the full potential of SERS can be achieved and the technique can be adopted for more routine use.
